# Supplementary material for: Single-Cell Sequencing of iPSC-Dopamine Neurons Reconstructs Disease Progression and Identifies HDAC4 as a Regulator of Parkinson Cell Phenotypes
Source: Cell Stem Cell. 2019 Jan 3;24(1):93–106.e6. doi: 10.1016/j.stem.2018.10.023 (PMC6327112; doi:10.1016/j.stem.2018.10.023)
Supplement: Document S1. Figures S1–S7 and Tables S1 and S2 [file mmc1.pdf]

Cell Stem Cell, Volume *24*

Supplemental Information

Single-Cell Sequencing of iPSC-Dopamine Neurons Reconstructs Disease Progression and Identifies HDAC4 as a Regulator of Parkinson Cell Phenotypes

Charmaine Lang, Kieran R. Campbell, Brent J. Ryan, Phillippa Carling, Moustafa Attar, Jane Vowles, Olga V. Perestenko, Rory Bowden, Fahd Baig, Meike Kasten, Michele T. Hu, Sally A. Cowley, Caleb Webber, and Richard Wade-Martins

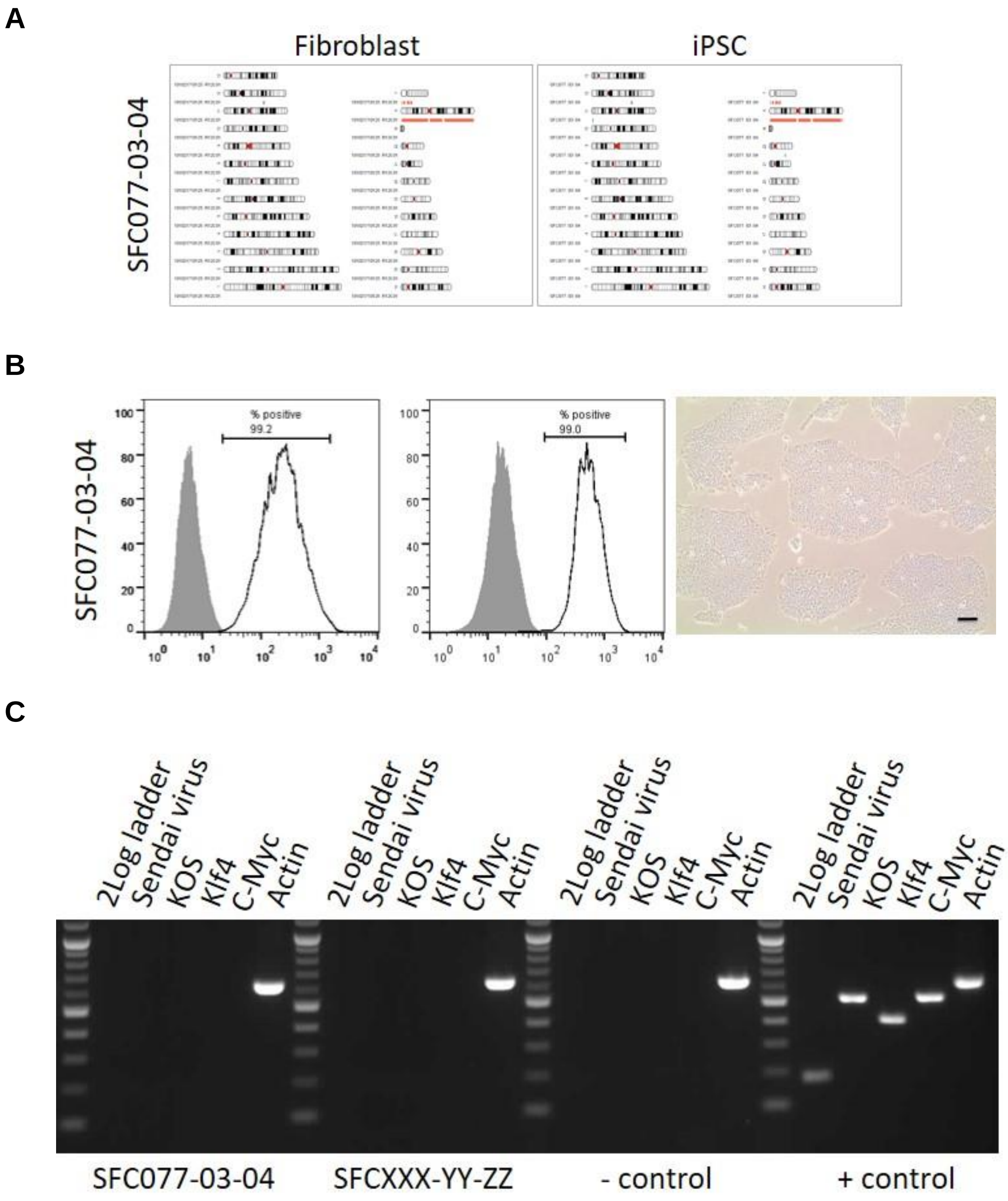

Supplementary Figure 1. Quality control (QC) of iPSC lines used in this study (related to STAR methods section Culture, reprogramming and characterisation of primary fibroblasts).
Representative iPSC QC of line SFC077-03-04 **(A)** Genome integrity was assessed by Illumina Human CytoSNP-12v2.1 or OmniExpress24SNP array and karyograms produced using KaryoStudio software (Illumina). Amplifications (green)/deletions (orange)/LOH regions (grey) are shown alongside the relevant chromosome (NB single-copy sex chromosomes are annotated orange). **(B)** FACS analysis confirmed expression of pluripotency markers Tra-1-60 and Nanog in iPSCs; open black plot represents antibody, filled grey plot represents isotype control; Right-hand panel shows expected iPSC colony morphology by phase-contrast microscopy. Scale bar = 100 µm. **(C)** Clearance of Cytotune Sendai vectors from iPSC lines: top panel Cytotune 1 (Log2 ladder; Sendai backbone 181 bp; Sox2 451 bp; Klf4 410 bp; c-myc 532 bp; Oct-4 483 bp; β-actin control 92 bp; + control, fibroblasts infected with Cytotune 5 days previously); lower panels Cytotune 2 (as per Cytotune 1 PCR products, but with KOS 528bp, β-actin control 623 bp).
All previously unpublished lines used in this paper have been deposited in the European Bank for induced pluripotent Stem Cells, EBiSC, https://cells.ebisc.org/ and are listed in the Human pluripotent stem cell registry, hPSCreg, https://hpscreg.eu/ along with their QC data.

A

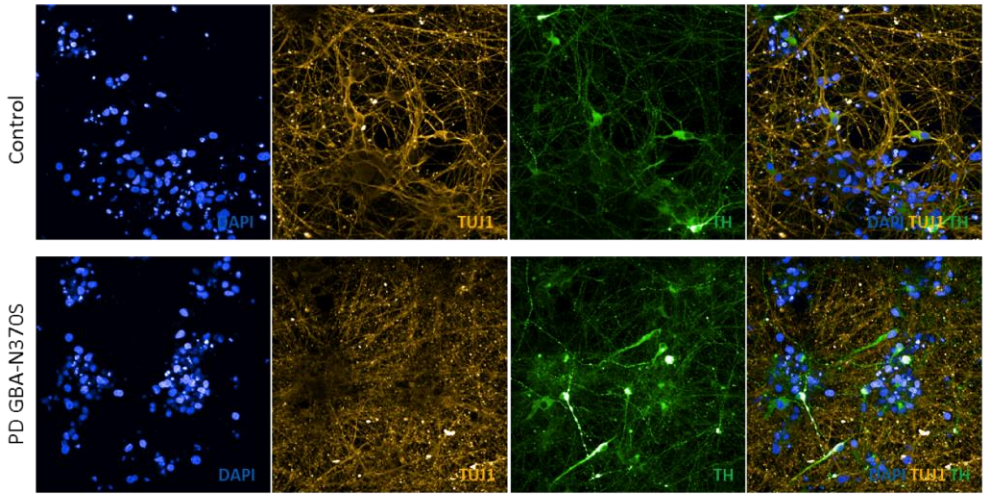

B

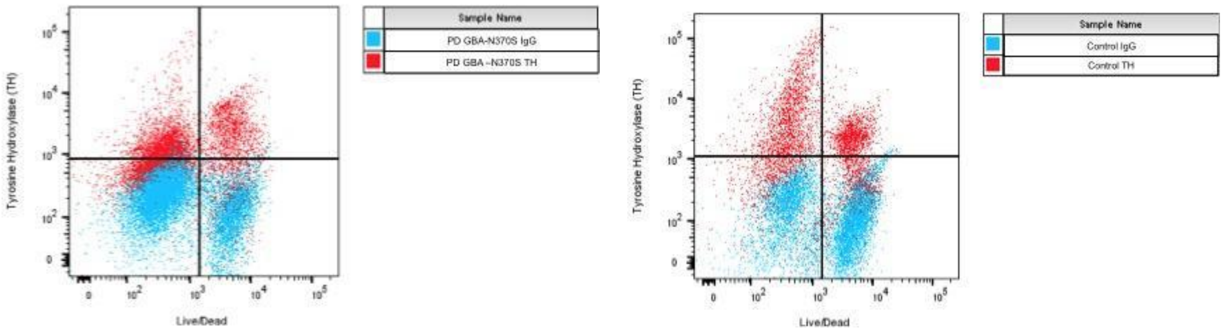

C

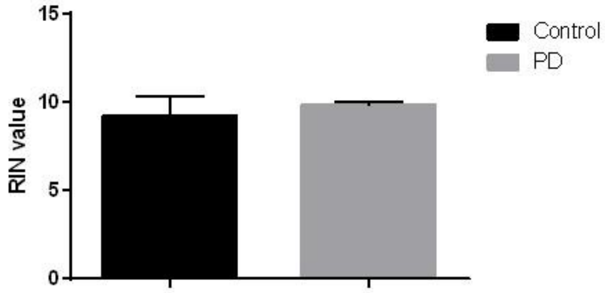

D

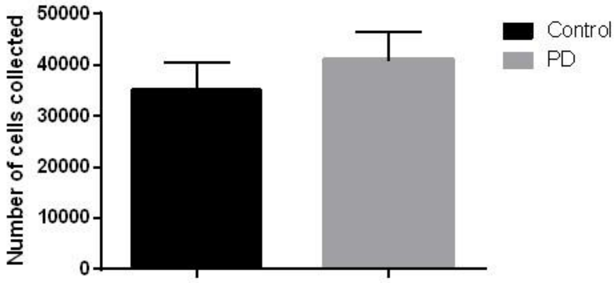

Supplementary Figure 2. Immunofluorescence, fluorescence activated cell sorting (FACS) and RNA integrity (RIN) values of Control and PD *GBA-N370S* samples (related to STAR methods section Generation and characterisation of iPSC derived dopamine neurons).
(A) Representative images of the successful differentiation of control and PD *GBA-N370S* dopaminergic neurons, displaying the nuclear marker (DAPI), neuronal marker (TUJ1) and the dopaminergic marker (TH). **(B)** Representative FACS plots of sorted TH positive neurons from Control and PD *GBA-N370S* samples. IgG2a was used as a FACS antibody control. **(C)** Number of TH+ cells collected from 3 control and 3 PD *GBA-N370S* samples. **(D)** RIN values of bulk sorted RNA from 3 control and 3 PD *GBA-N370S* samples.

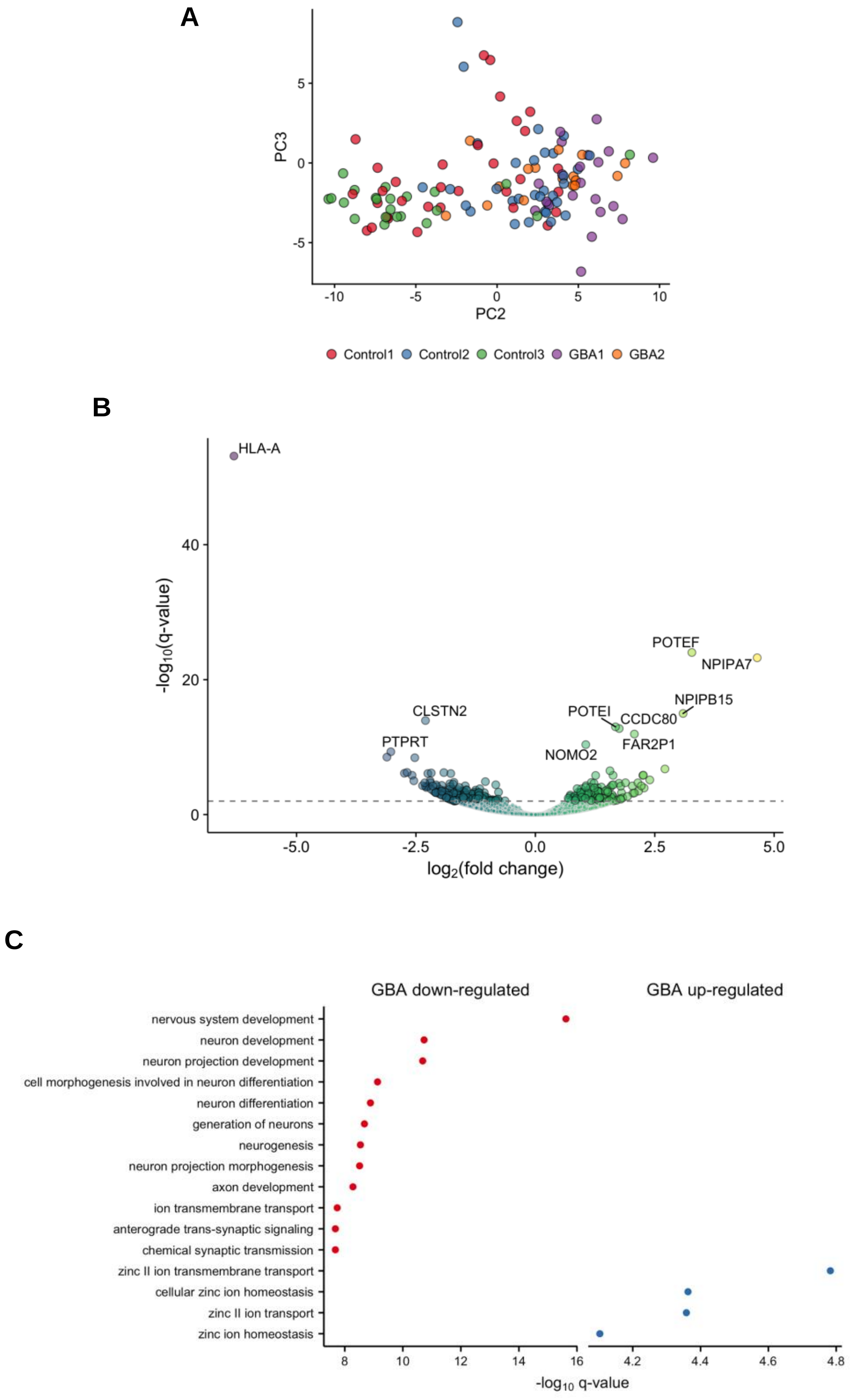

Supplementary Figure 3. Bulk and single DE genes analysis with the removal of GBA3 (related to Figure 2).

(A) Transcriptome PCA analysis after the removal of GBA3, resolving the remaining two PD *GBA-N370S* PD patients and three controls. **(B)** Volcano plot of the differential expression analysis using DESeq2 identified 310 genes (FDR 1%) differentially expressed between case-control with the removal of GBA3 **(C)** A GO enrichment analysis of the up and down-regulated genes in PD *GBA-N370S* patients, with the removal of GBA3, highlights important pathways involved in zinc homeostasis and neuronal function respectively.

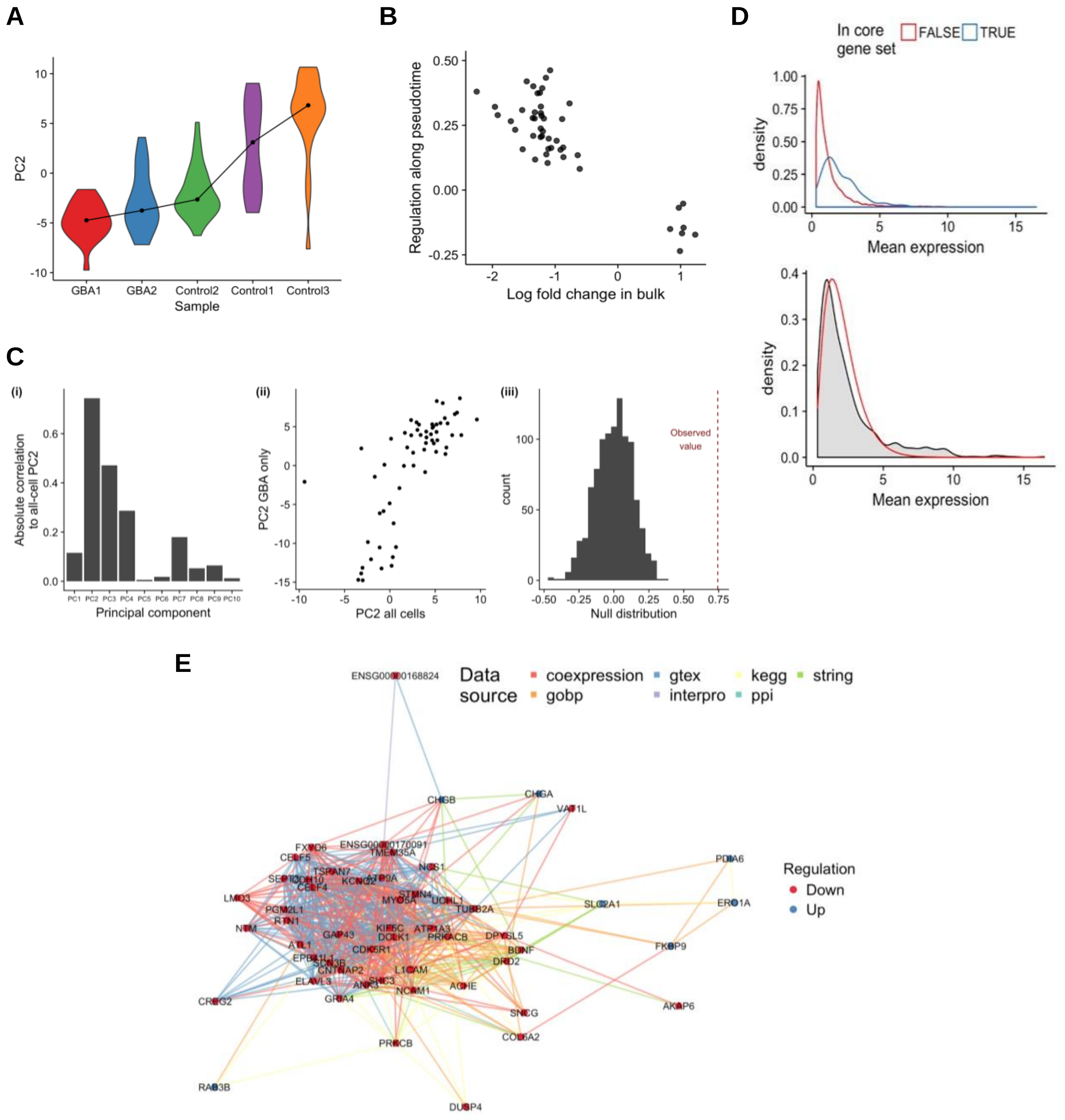

Supplementary Figure 4. Single-cell trajectory analysis recapitulates a case-control ordering of cells and Bulk and single cell RNA-seq combined gene set analysis (related to Figure 3).
(A) Violin plot showing principal component 2 (PC2) scores for each sample after the removal of GBA3. PC2 roughly segregates cells into case/control status and thus may be seen to represent the continuous transcriptional signature of cells from control to GBA status. **(B)** The regulation along pseudotime (PC2) as provided by *switchde* show consistent regulation with bulk DE analysis for the core gene set identified in both single and bulk DE analysis along with SC3 clustering. **(C-i)** PC2 of the data is equivalent in GBA samples alone, demonstrating the control to disease transition heterogeneity exists within the disease samples. **(C-ii)** The correlation of PC2 using GBA cells only compared to the top 10 PC's using all cells reveals high correlation with PC2 (rho = 0.74), exemplified in a scatter plot. **(C-iii)** By randomly permuting cells, performing PCA and comparing correlations to PC2, a null distribution was created, the observed correlation $p < 0.001$. **(D)** Mean expression of genes in the core gene set compared to whole transcriptome and compared to Gamma distribution fit (red). **(E)** A phenotypic linkage network (PLN) representing functional similarity between the core set of genes identified in both bulk and single-cell RNA-seq.

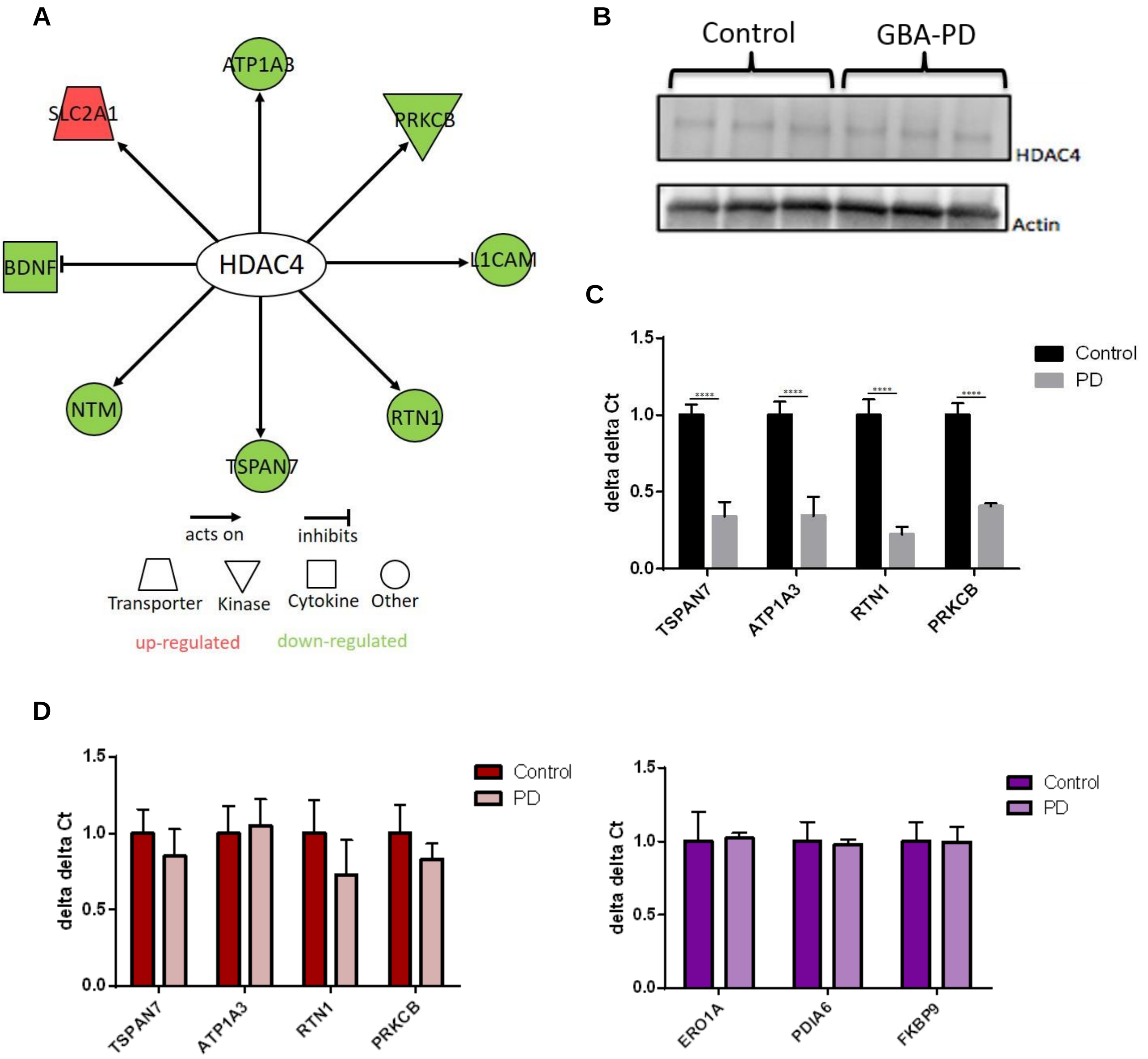

Supplementary Figure 5. HDAC4 as a master regulator of a set of down-regulated genes in PD *GBA-N370S* patients (related to Figure 3).
(A) IPA (Qiagen) identified HDAC4 as an upstream regulator of a number of genes identified as down-regulate and one up-regulated gene, in the core set of 60 DE genes (modified from IPA Qiagen). **(B)** HDAC4 protein levels are unchanged in control and PD *GBA-N370S* patients. **(C)** The four HDAC4 controlled genes in the core set of genes identified in both bulk and single cell RNA-seq were confirmed as down-regulated in PD *GBA-N370S* patients compared to controls by qRT-CPR (****p<0.0001). **(D)** No difference in the expression of HDAC4 controlled genes (*TSPAN7*, *ATP1A3*, *RTN1* and *PRKCB*) and ER stress genes (*ERO1A*, *PDIA6* and *FKBP9*) identified in the case-control axis, in induced pluripotent stem cells pre-differentiation.

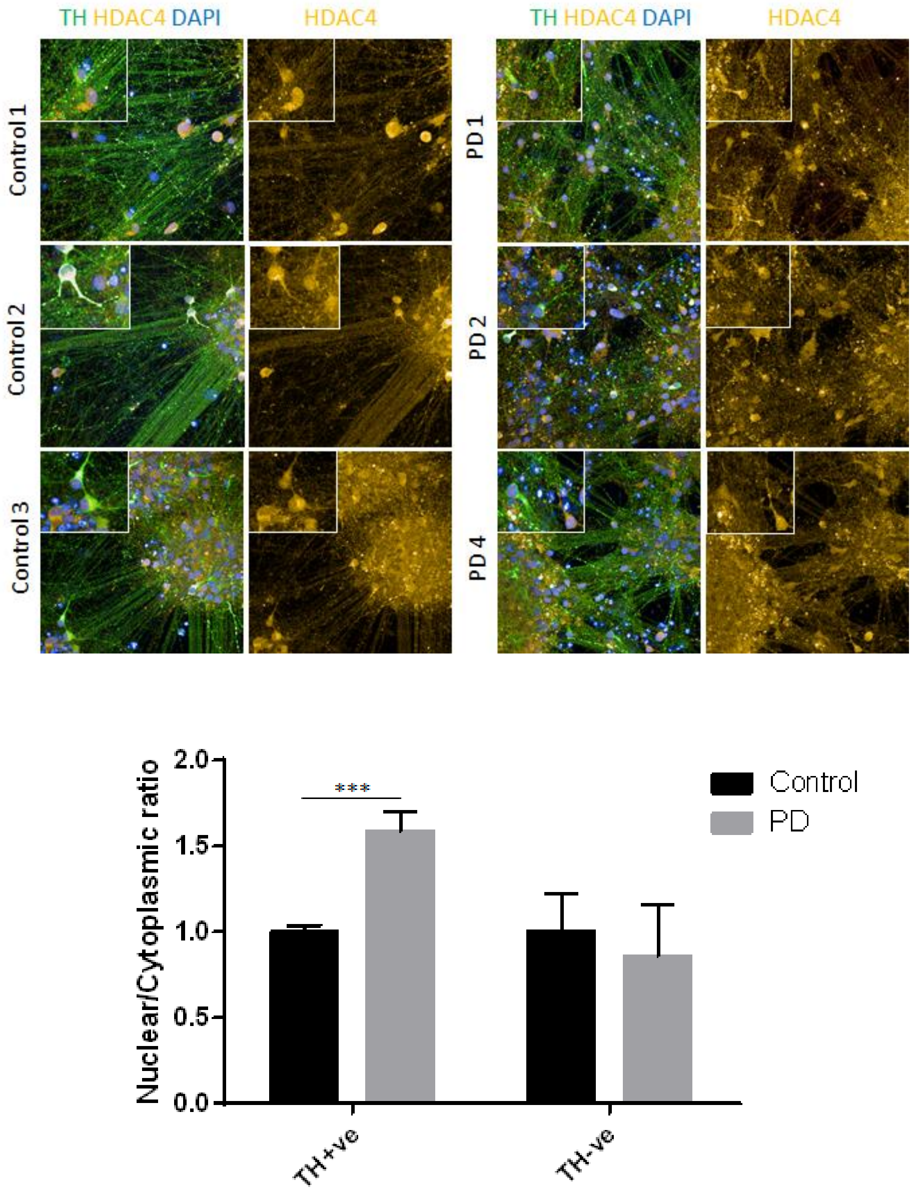

Supplementary Figure 6. HDAC4 nuclear vs cytoplasmic ratio in non-dopaminergic neurons in PD *GBA-N370S* patients and controls (related to Figure 4).

No difference in HDAC4 nuclear/cytoplasmic ratio intensity in non-TH positive neurons in three control and three PD *GBA-N370S* patient lines. Data represented as mean ± SEM ***p<0.001 of three control and three PD *GBA-N370S* patients.

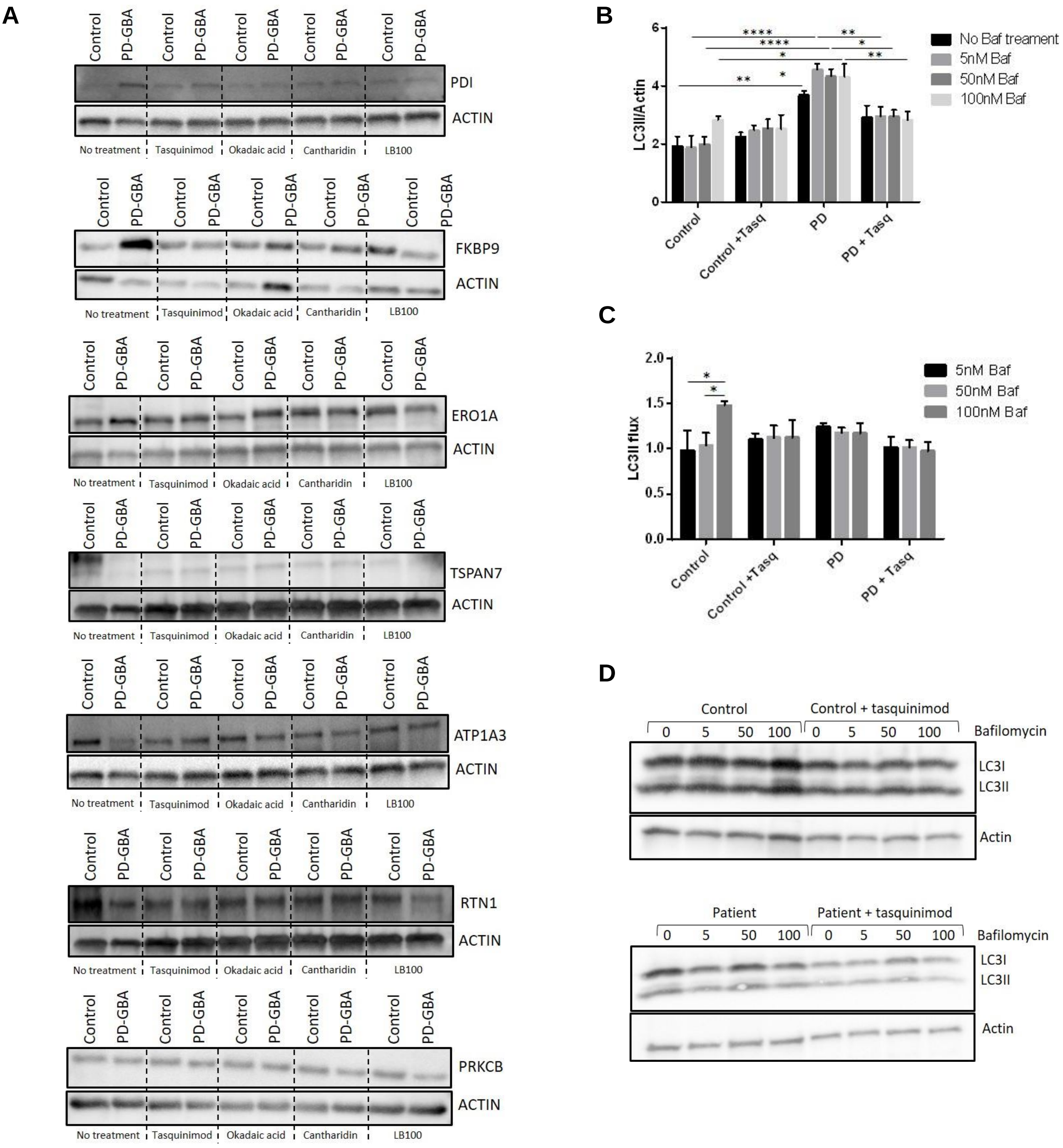

Supplementary Figure 7. Representative western blot images of the protein graphs in Figure 5 and autophagic flux in control and PD *GBA-N370S* patient iPSC-derived dopamine neurons with and without tasquinimod treatment (related to Figure's 5 and 6).

(A) Western blots display an increase in ER stress proteins (*PDI*, *FKBP9* and *ERO1A*) and a decrease in HDAC4 related proteins (*TSPAN7*, *ATP1A3*, *RTN1* and *PRKCB*) in PD *GBA-N370S* patients compared to controls. The addition of the HDAC4 modulating drugs (Tasquinimod, Okadaic acid, Cantharidin and LB100) mostly revert these phenotypes. **(B)** LC3II/Actin levels in control and PD *GBA* neurons +/- tasquinimod and 0, 5, 50 and 100 nM bafilomycin. Black bars (no bafilomycin treatment) and grey bars (with bafilomycin treatment). **(C)** Data in Figure 1A represented as autophagic flux, quantified by dividing levels of LC3-II with bafilomycin treatment by the level of LC3-II without treatment. **(D)** Representative western blots for LC3-II and actin in control and PD *GBA* neurons, compared to a calibration sample. Data represented as mean ± SEM (*p<0.05, **p<0.01 and ****p<0.0001) of three control and three PD *GBA-N370S* patients.

Supplementary Table 1. Control, PD *GBA-N370S* and Idiopathic PD lines used in this study (related to STAR methods section iPSC lines and participation recruitment and subject details table).

Donor ID	iPSC clone	Study ID	Genotype	Age	Characterisation
AH016	03/06	Control 1	wt/wt	80 M	Sandor et al 2017
JR053	06/01	Control 2	wt/wt	68 M	This study
OX1<br>SFC841-03	18/19<br>01/02	Control 3	wt/wt	36 M	Van Wilgenburg et al 2013
SFC156-03	01	Control 4	wt/wt	75 M	This study
SFC840-03	06	Control 5	wt/wt	67 F	Fernandes et al 2016
SFC067-03	01	Control 6	wt/wt	72 M	This study
MK088	01	GBA 1	N370S/wt	46 M	Fernandes et al 2016
MK071	03	GBA 2	N370S/wt	81 F	Fernandes et al 2016
SFC834-03	03	GBA 3	N370S/wt	72 M	Fernandes et al 2016
MK082	26	GBA 4	N370S/wt	51 M	This study
SFC077-03	04	Idiopathic PD 1	N/A	65 M	This study
SFC844-03	12	Idiopathic PD 2	N/A	72 M	This study
SFC120-03	04	Idiopathic PD 3	N/A	72 M	This study
SFC865-03	07	Idiopathic PD 4	N/A	69 M	This study

Supplementary Table 2. QRT-PCR primers used in this study (related to STAR methods section qRT-PCR, immunocytochemistry and western blot).

Primer name	Primer sequence	Source
B2M F	TTCTGGCCTGGAGGCTATC	This paper
B2M R	TCAGGCAATTTGACTTTCCATTC	This paper
FKBP9 F	AGCTTGCCTACGGAAATGAA	This paper
FKBP9 R	GGGGCTTGAAATAGGTGTGA	This paper
ERO1A F	AATGTCGTCTGTGGGGAAAG	This paper
ERO1A R	AGGTCCACTTTCTGGCATATTT	This paper
PDIA6 F	CAGAATGGAAGAAAGCAGCA	This paper
PDIA6 R	TCCCTGAACACCATACTGACC	This paper
TSPAN7 F	TGTTGTCTTTGGCCTGTTTG	This paper
TSPAN7 R	TGACGAAACACAAACCCTGA	This paper
ATP1A3 F	CCTTGGAGACTCGGAACATC	This paper
ATP1A3 R	GATACGGCCCATGACAGTG	This paper
RTN1 F	AGTGCAGAAAACCGACGAAG	This paper
RTN1 R	GGAAGAGCCTCCTCAGTTCC	This paper
PRKCB F	TACTCCAGCCCCACGTTTTG	This paper
PRKCB R	TCACTTCCTTCTGGTGGCAC	This paper
RPS12 F	TGCGTTCAAGATTCAACTTCAC	This paper
RPS12 R	TGAGGGCAGTCTTCAGAACC	This paper
RPS17 F	CATTATCCCCAGCAAAAAGC	This paper
RPS17 R	CCTCTTACTGGGCCTCTCTG	This paper
RPS6 F	CGATGAACGCAAACTTCGTA	This paper
RPS6 R	ACCACTGATTCGGACCACAT	This paper
pMXsAS3200v2	TTATCGTCGACCACTGTGCTGGCG	(Takahashi et al., 2007)
mNanog	GCTCCATAACTTCGGGGAGG	(Takahashi et al., 2007)
