# Supplementary material for: Single-Cell Sequencing of iPSC-Dopamine Neurons Reconstructs Disease Progression and Identifies HDAC4 as a Regulator of Parkinson Cell Phenotypes
Source: Cell Stem Cell. 2019 Jan 3;24(1):93–106.e6. doi: 10.1016/j.stem.2018.10.023 (PMC6327112; doi:10.1016/j.stem.2018.10.023)
Supplement: Document S2. Article plus Supplemental Information [file mmc2.pdf]

Article

Cell Stem Cell

Single-Cell Sequencing of iPSC-Dopamine Neurons Reconstructs Disease Progression and Identifies HDAC4 as a Regulator of Parkinson Cell Phenotypes

Graphical Abstract

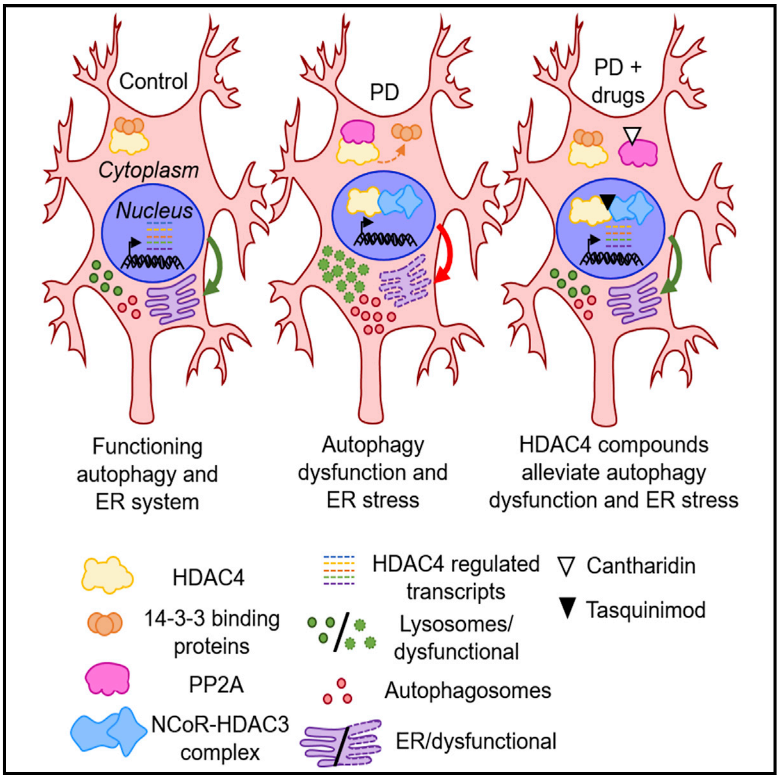

Authors

Charmaine Lang, Kieran R. Campbell, Brent J. Ryan, ..., Sally A. Cowley, Caleb Webber, Richard Wade-Martins

Correspondence

webberc4@cardiff.ac.uk (C.W.), richard.wade-martins@dpag.ox.ac.uk (R.W.-M.)

In Brief

Bulk and single-cell RNA-seq of iPSC-derived dopamine neurons from control and PD *GBA-N370S* patients stratified a clinically distinct patient and revealed HDAC4 as a potential therapeutic PD target. Pharmacological modulation of HDAC4 rescued PD-related phenotypes in *GBA-N370S* neurons. HDAC4 perturbation was also observed in a subset of sporadic PD patients.

Highlights

- Single-cell RNA-seq stratifies patients with similar clinical presentation
- A pseudotemporal profile aligns single cells along a control to disease axis
- HDAC4 is mislocalized to the nucleus in PD patient iPSC-derived dopamine neurons
- Repurposed compounds correct HDAC4 mislocalization and revert PD-related phenotypes

Lang et al., 2019, Cell Stem Cell *24*, 93–106
January 3, 2019 © 2018 The Authors. Published by Elsevier Inc.
https://doi.org/10.1016/j.stem.2018.10.023

Cell Stem Cell
Article

Single-Cell Sequencing of iPSC-Dopamine Neurons Reconstructs Disease Progression and Identifies HDAC4 as a Regulator of Parkinson Cell Phenotypes

Charmaine Lang,[1,8] Kieran R. Campbell,[1,2,6,8] Brent J. Ryan,[1] Phillippa Carling,[1] Moustafa Attar,[2] Jane Vowles,[3] Olga V. Perestenko,[3] Rory Bowden,[2] Fahd Baig,[4] Meike Kasten,[5] Michele T. Hu,[4] Sally A. Cowley,[3] Caleb Webber,[1,7,*] and Richard Wade-Martins[1,9,*]
[1]Oxford Parkinson's Disease Centre, Department of Physiology, Anatomy and Genetics, University of Oxford, South Parks Road, Oxford, UK
[2]The Wellcome Centre for Human Genetics, University of Oxford, Oxford, UK
[3]Sir William Dunn School of Pathology, University of Oxford, South Parks Road, Oxford OX1 3RE, UK
[4]Oxford Parkinson's Disease Centre, Nuffield Department of Clinical Neurosciences, University of Oxford, Oxford, UK
[5]Department of Psychiatry and Psychotherapy and Institute of Neurogenetics, University of Lübeck, Lübeck, Germany
[6]Present address: Department of Statistics, University of British Columbia, Vancouver, BC, Canada
[7]Present address: Dementia Research Institute, Hayden-Ellis Building, Cardiff University, Maindy Road, Cardiff, UK
[8]These authors contributed equally
[9]Lead Contact
*Correspondence: webberc4@cardiff.ac.uk (C.W.), richard.wade-martins@dpag.ox.ac.uk (R.W.-M.)
https://doi.org/10.1016/j.stem.2018.10.023

SUMMARY

Induced pluripotent stem cell (iPSC)-derived dopamine neurons provide an opportunity to model Parkinson's disease (PD), but neuronal cultures are confounded by asynchronous and heterogeneous appearance of disease phenotypes *in vitro*. Using high-resolution, single-cell transcriptomic analyses of iPSC-derived dopamine neurons carrying the *GBA-N370S* PD risk variant, we identified a progressive axis of gene expression variation leading to endoplasmic reticulum stress. Pseudotime analysis of genes differentially expressed (DE) along this axis identified the transcriptional repressor histone deacetylase 4 (HDAC4) as an upstream regulator of disease progression. HDAC4 was mislocalized to the nucleus in PD iPSC-derived dopamine neurons and repressed genes early in the disease axis, leading to late deficits in protein homeostasis. Treatment of iPSC-derived dopamine neurons with HDAC4-modulating compounds upregulated genes early in the DE axis and corrected PD-related cellular phenotypes. Our study demonstrates how single-cell transcriptomics can exploit cellular heterogeneity to reveal disease mechanisms and identify therapeutic targets.

INTRODUCTION

Parkinson's disease (PD) is a neurodegenerative disorder affecting over 6 million people worldwide, predominantly over the age of 65 (Baker and Graham, 2004). PD is characterized by motor symptoms, including rigidity, resting tremor, bradykinesia, and postural instability, and non-motor features, including cognitive impairments, anxiety, and depression (Gonera et al., 1997). The motor symptoms are due to the progressive loss of dopamine neurons in the substantia nigra pars compacta, with approximately 50% of dopamine neurons lost in the midbrain at the onset of motor symptoms (Fearnley and Lees, 1991). The majority of PD cases are idiopathic, with only about 10% attributed to inherited PD cases. The glucocerebrosidase gene encodes the lysosomal enzyme, GCase, homozygous mutations in which cause the autosomal recessive lysosomal storage disorder, Gaucher's disease (GD) (Hruska et al., 2008). *GBA* was first found to be associated with PD due to a high incidence of PD in both GD patients and heterozygous *GBA* carrier family members (Tayebi et al., 2003). Approximately 5%–10% of PD patients carry a heterozygous *GBA* mutation, making *GBA* variants the most common genetic risk factors for PD. The *GBA-N370S* mutation is the most common *GBA* risk variant, and patients have a clinical presentation similar to idiopathic PD (Beavan and Schapira, 2013).

Understanding the molecular basis of neurodegenerative disease has been hindered by the inaccessibility of live vulnerable human neurons from patients. The advent of induced pluripotent stem cell (iPSC) technology enables the study of patient-derived dopamine neurons from PD patients retaining genetic risk variants. Work with iPSC-derived dopamine neurons from PD patients carrying *GBA* (Fernandes et al., 2016; Schöndorf et al., 2014) or leucine-rich repeat kinase 2 (*LRRK2*) mutations (Sánchez-Danés et al., 2012) has revealed deficits in protein homeostasis via the endoplasmic reticulum (ER), autophagic, and lysosomal pathways. However, iPSC-derived neuronal cultures often contain cellular heterogeneity, confounding gene expression profiling of a specific cell type.

Recently, we developed a method to obtain pure populations of iPSC-derived dopamine neurons by fluorescence-activated cell sorting (FACS), which we used to profile gene expression in PD *LRRK2-G2019S* iPSC-derived dopamine neurons (Sandor et al., 2017). Nonetheless, cellular heterogeneity remains, even within a purified population, as individual cells are unlikely to

© 2018 The Authors. Published by Elsevier Inc.
This is an open access article under the CC BY license (http://creativecommons.org/licenses/by/4.0/).

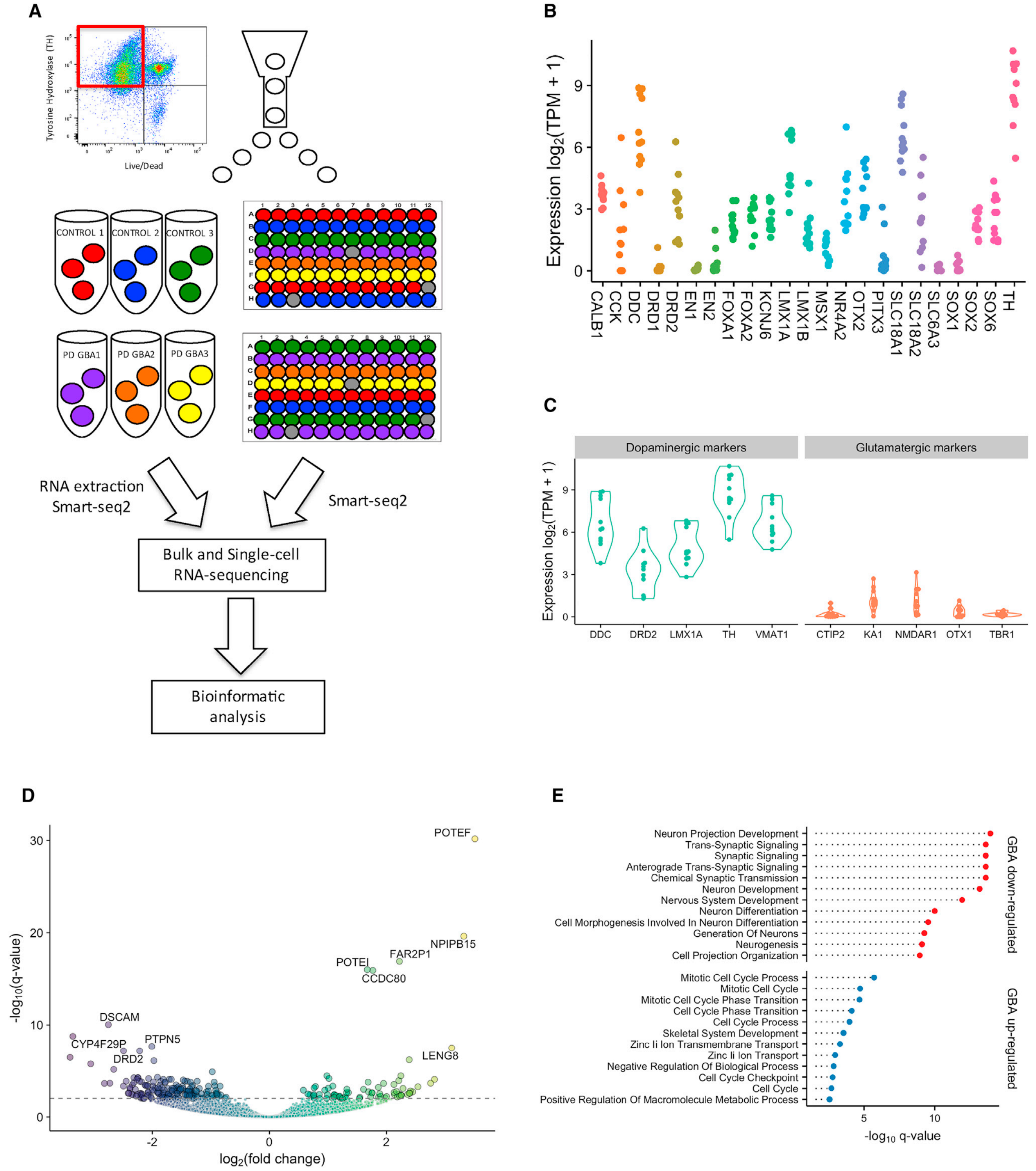

Figure 1. Bulk RNA-Seq Analysis Confirms Purification of iPSC-Derived Dopamine Neurons and Identifies 247 DE Genes between Control and PD *GBA-N370S* Patients Enriched for Genes in Pathways of Neuronal Function

(A) Schematic of sorting the tyrosine hydroxylase-positive (TH+) iPSC-derived dopamine neurons from three controls and three PD *GBA-N370S* patients displaying a FACS plot identifying live/TH+ cells for sorting into bulk collection and into 96-well plates for single-cell RNA sequencing (gray wells indicate blank wells). Bulk and single cells went through RNA extraction, cDNA synthesis, and amplification before undergoing sequencing and bioinformatic analysis.

(legend continued on next page)

experience the same gene-driven perturbation synchronously. Bulk gene expression profiling across thousands of cells provides only a population average, obscuring that cells may be at different points in one or more disease-relevant processes. By contrast, profiling gene expression within individual cells can exploit population heterogeneity, distinguishing distinct cell subpopulations and discerning the progression of cells through the disease-relevant processes being modeled (Reid and Wernisch, 2016). Our FACS-based purification method for dopamine neurons is readily applicable to plate-based deep single-cell profiling (Picelli et al., 2013).

Here, we applied bulk and deep single-cell gene expression profiling to purified populations of iPSC-derived dopamine neurons from three PD patients carrying the *GBA-N370S* variant. Unique to a single *GBA-N370S* patient, we identified increased activation of the signal recognition particle pathway. This molecular stratification was validated by clinical follow-up, which confirmed a revised diagnosis of progressive supranuclear palsy for that patient, who was removed from further downstream analysis.

Combining bulk and single-cell expression profiles, we identified a robust set of 60 genes whose expression captured an axis of variation between cells from controls and the remaining two PD *GBA-N370S* patients. Aligning individual cells across this axis generated a pseudotemporal profile along which the sequence of changes in the expression of individual genes could be inferred. Although variation in gene expression at the end of the pseudotemporal profile was associated with an increase in ER stress, previously characterized in PD, many early differentially expressed (DE) genes were found to be downregulated by histone deacetylase 4 (HDAC4), a class IIa histone deacetylase, which acts as a transcriptional repressor that shuttles between the nucleus and the cytoplasm. HDAC4 was found to be mislocalized to the nucleus in PD *GBA-N370S* iPSC-derived dopamine neurons. Modulation of HDAC4 activity or localization reversed the downregulation of the core set of DE genes and ameliorated PD-related cellular phenotypes previously described in PD *GBA-N370S* dopamine neurons, including ER stress, autophagic and lysosomal perturbations, and increased α-synuclein release. Finally, we demonstrated HDAC4 mislocalization and perturbation of the same core set of DE genes in iPSC-derived dopamine neurons from a subset of idiopathic PD cases. Our work demonstrates how we can exploit cellular heterogeneity to reveal disease mechanisms and therapeutic targets.

RESULTS

Characterization and Purification of iPSC-Derived Dopamine Neurons by FACS

Previously, we reported that iPSC-derived dopamine neurons obtained from PD *GBA-N370S* patients exhibited increased ER stress, autophagic and lysosomal perturbations, and elevated α-synuclein release (Fernandes et al., 2016). To further investigate variation in gene expression, which may underlie disease processes, we sought to purify iPSC-derived dopamine neurons from control and *GBA-N370S* patients and subject them to both bulk and single-cell RNA sequencing (Figure 1A).

iPSC lines derived from three PD *GBA-N370S* patients and three controls (Figure S1) were differentiated into dopamine neurons, as previously (Kriks et al., 2011), with minor modifications (Beevers et al., 2017). All iPSC lines were successfully differentiated, typically yielding dopaminergic neuronal cultures 40%–60% positive for tyrosine hydroxylase (TH), a marker of dopamine neurons (Figure S2A). To isolate dopamine neurons from the heterogeneous population of differentiated cells, neurons were sorted by FACS as described (Sandor et al., 2017; Figure S2B). Approximately 35–40,000 TH+ neurons were purified and collected from each of the three control and three PD *GBA-N370S* samples, and RNA was extracted. There was no significant difference in the number of cells collected for each group (Figure S2C) or in extracted RNA quality by RNA integrity (RIN) values of ∼9 for the bulk-collected FACS-purified samples (Figure S2D).

Bulk RNA Sequencing of Purified iPSC-Derived Dopamine Neurons Reveals Downregulated Genes Associated with Synaptic Function and Development

Bulk RNA sequencing (RNA-seq) profiles of FACS-purified cells showed increased expression of dopamine neuron marker genes (Figure 1B), such as tyrosine hydroxylase (*TH*), dopa decarboxylase (*DDC*), solute carrier family 18 member A1 (*VMAT1*), LIM homeobox transcription factor 1 alpha (*LMX1A*), and dopamine receptor D2 (*DRD2*). Purified neurons lacked expression of glutamatergic neuronal markers, including COUP-TF-interacting protein 2 (*CTIP2*), N-methyl-D-aspartate receptor subunit NR1 (*NMDAR1*), orthodenticle homeobox 1 (*OTX1*), and T-box brain protein 1 (*TBR1*), confirming purification specifically of dopamine neurons (Figure 1C).

DE analysis between the PD *GBA-N370S* and control lines identified differences in gene expression patterns, with 247 genes DE at a 1% false discovery rate (FDR) (Figure 1D). Overall, gene ontology (GO) enrichment analysis of the upregulated and downregulated genes in PD *GBA-N370S* iPSC-derived dopamine neurons highlighted DE of genes involved in neuronal development, neuronal differentiation, and synaptic activity, whereas zinc ion transport functions featured in the upregulated genes (Du et al., 2017; Forsleff et al., 1999; Park et al., 2014; Figure 1E).

Single-Cell RNA-Seq Stratifies PD *GBA-N370S* Patients

Initial analyses of the 146 single-cell transcriptomic profiles passing quality control (QC) demonstrated the same enrichment of neuronal marker genes as the bulk transcriptional profiles, although with individual cell gene dropouts typical of single-cell data (Pierson and Yau, 2015; data not shown). Principal component analysis (PCA) found that the transcriptional profiles of the cells segregated by patient origin along both the second and third components (Figure 2A). Notably, cellular transcriptional variation attributed to dopamine neurons derived from one of

(B and C) Expression of dopamine neuron-specific markers (B) and the absence of glutamatergic markers (C) in the purified bulk iPSC-derived dopamine neurons.
(D) Volcano plot showing 247 genes DE between *GBA-N370S* PD versus control identified by DESeq2 (FDR 1%).
(E) GO enrichment analysis of the upregulated and downregulated genes in PD *GBA-N370S* patients highlights DE of genes involved in neuronal development and synaptic activity.

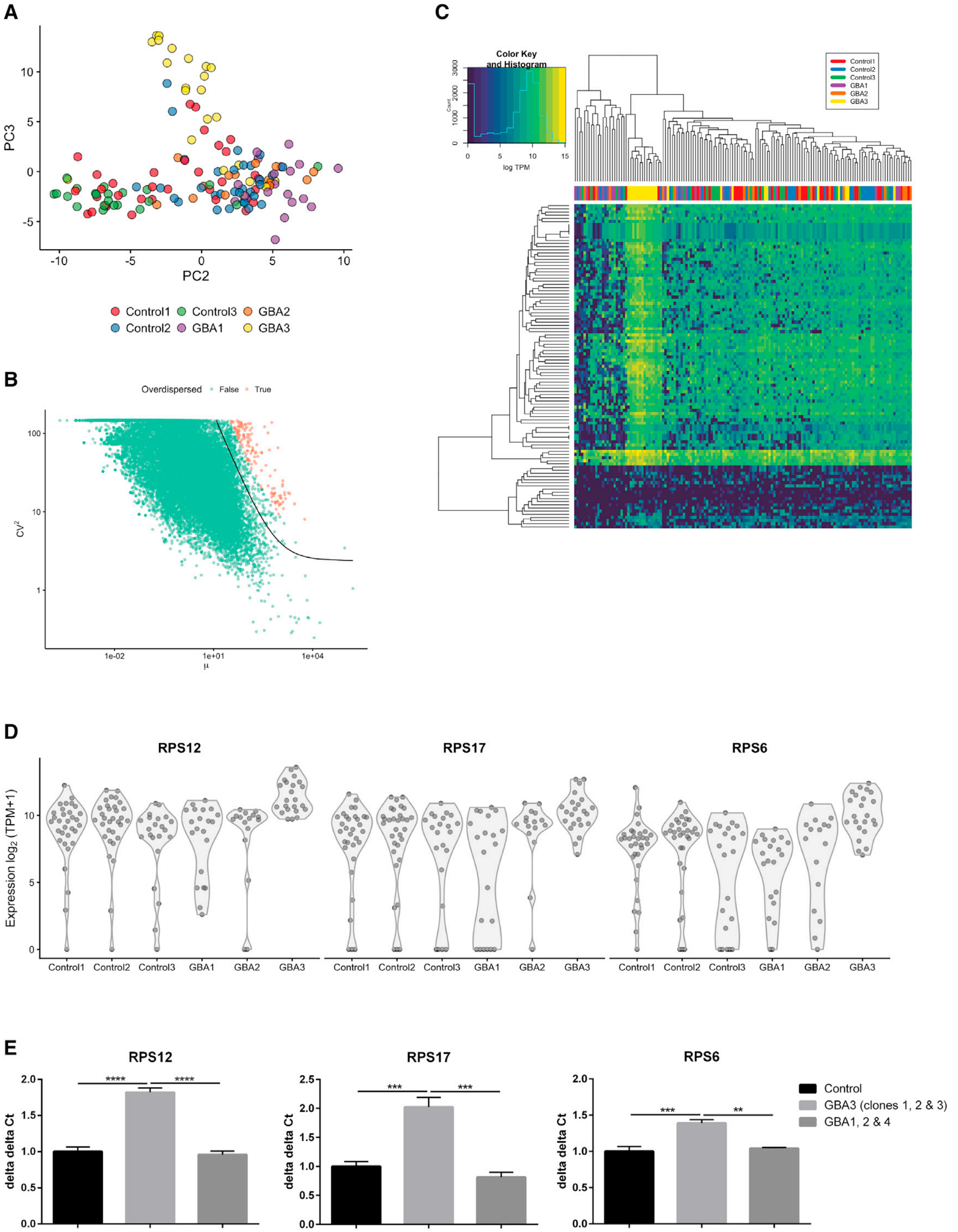

Figure 2. Single-Cell RNA-Seq Stratification Identifies iPSC-Derived Dopamine Neurons from GBA3 as Significantly Different from Both PD Patient and Control Neurons

(A) Transcriptome PCA analysis resolves GBA3 neurons (yellow) from the remaining two PD *GBA-N370S* patients and three controls.

(B) Over-dispersion analysis identifies a subset of genes that vary more than expected due to technical fluctuations in the dataset alone.

(C) Heatmap of the single-cell RNA-seq samples identifies an enrichment in the endoplasmic reticulum (ER) signal recognition particle (SRP) pathway in GBA3.

(D) Expression in $\log_2$ (TPM+1) of three genes (*RPS12*, *RPS17*, and *RPS6*) prioritized from those significantly DE within the SRP pathway between GBA3 and controls 1, 2, and 3 and GBA1 and 2. DE analysis was performed using a two-sided Wilcoxon signed-rank test on all genes in the SRP pathway.

(E) The upregulation of the three selected genes involved in this pathway was confirmed in iPSC-derived dopamine neurons differentiated from three iPSC clones of GBA3 compared to the three original controls and two PD *GBA-N370S* patients (GBA1 and 2), plus a fourth PD *GBA-N370S* patient (GBA4). Data are represented as mean ± SD (**$p < 0.01$; ***$p < 0.001$; ****$p < 0.0001$).

the PD *GBA-N370S* patients (referred to as "GBA3") was represented by the third principal component (Figure 2A). Overdispersion analysis used to identify genes that varied more than can be expected due to the inherent technical variation in the dataset (Brennecke et al., 2013) observed 143 genes (0.6%) as significantly over-dispersed at 5% FDR (Figure 2B). A GO enrichment analysis on the over-dispersed genes identified the signal recognition particle (SRP)-dependent co-translational protein targeting to membrane pathway and related processes as driving this variation.

The separation of GBA3 dopamine neurons along the third principal component, and the large variation in a small set of genes belonging to one pathway, prompted concerns that a single sample could be driving the variation in gene expression between the PD *GBA-N370S* cases and controls. The expression of genes belonging to the SRP-dependent co-translational protein targeting to membrane pathway strikingly demonstrated increased activation in the GBA3 patient alone, who clustered apart from all other case and control samples (Figure 2C). Expression analysis of three genes in this pathway—ribosomal protein S12 (*RPS12*), ribosomal protein S17 (*RPS17*), and ribosomal protein S6 (*RPS6*)—confirmed the upregulation in this pathway to be specific to patient GBA3 (Figure 2D).

Expression of the same three genes was confirmed by qRT-PCR in iPSC-derived dopamine neurons generated from the original GBA3 iPSC line, two further iPSC lines from the GBA3 patient, the three controls, the two other original GBA patients, and an additional fourth PD *GBA-N370S* patient (GBA4; Figure 2E). Comparison of the three GBA3 patient lines with the three controls and GBA patients 1, 2, and 4 confirmed elevation of the SRP-dependent co-translational protein targeting to membrane pathway to be specific to GBA3, proposing a molecular stratification of the patients used in this study.

Although all the patients in our study fulfilled UK Brain Bank diagnostic criteria for clinically probable PD at presentation, longitudinal clinical follow-up allows the diagnosis to be reviewed in light of disease progression and subsequent medication response. GBA patients 1, 2, and 4 presented at an early stage with akinetic-rigid parkinsonism and maintained a good levodopa response for their first five years of treatment without significant falls or dementia. In contrast, patient GBA3 presented with akinetic-rigid parkinsonism, failed to respond to standard medication (600 mg levodopa with benserazide 150 mg daily), and presented with early dementia and frequent falls two years after initial PD diagnosis. A supranuclear gaze palsy with dysarthria was then noted, and the patient received a revised clinical diagnosis of progressive supranuclear palsy (PSP). The stratification of PSP from PD among these *GBA-N370S* carriers by single-cell profiling of their iPSC-derived dopamine neurons is therefore consistent with the clinical stratification and reveals a potential disease-relevant pathway for PSP.

A Functionally Enriched Gene Set Defines a Pseudotemporal Axis of PD *GBA-N370S* iPSC-Derived Dopamine Neuron Gene Expression Variation

Upon removal of GBA3 from the analysis, we observed minimal changes in the set of genes found to be DE (Figure S3). Analysis of the transcriptomes of individual dopamine neurons broadly segregated along the second principal component from a higher concentration of control cells to a higher concentration of PD *GBA-N370S* case cells (Figure S4A). We hypothesized that the case-control divergence along this component reflected cells that were at varying points in a common disease-related process, with GBA1 and GBA2 neurons that were more control-like being at an earlier point in the same process than GBA1 and GBA2 dopamine neurons that were less control-like. Our approach is comparable to the idea of "pseudotime" in the context of cellular differentiation (Haghverdi et al., 2016; Ji and Ji, 2016; Reid and Wernisch, 2016).

As cells segregated by case-control status along the second principal component, there was a possibility the data simply represented two distinct cell types with the apparent continuum due to transcriptional noise. To test this hypothesis, we repeated principal-component analysis on the *GBA-N370S* iPSC-derived dopamine neurons alone and found a remarkable correlation of the second principal component when all cells are included (Figure S4C). Therefore, the transcriptional heterogeneity at the single-cell level represents a continuous disease axis from case to control.

We next sought to identify a core set of genes consistently perturbed across both bulk RNA-seq and the single-cell transcriptomic signature. This core set was identified as the intersection of two gene sets from the analysis: (1) those DE in bulk RNA-seq using DESeq2 after the removal of GBA3 (at 5% FDR) and (2) those DE across PC2 using *switchde* (at 5% FDR; Campbell and Yau, 2017; Figure S4B). We further refined this set to include only those additionally identified as discriminating marker genes after clustering the single-cell RNA-seq data using SC3 (Kiselev et al., 2017). By combining genes found through both bulk and single-cell DE as well as single-cell clustering (STAR Methods), we identified a core set of 60 genes, 52 of which were consistently downregulated and 8 of which were consistently upregulated in PD *GBA-N370S* iPSC-derived dopamine neurons (Figure S4B).

To validate that our core set of 60 genes were functionally convergent, we assessed the functional similarity between these genes within a phenotypic linkage network, as compared to known PD genes and a random background set controlled for the relevant core set gene properties (STAR Methods; Figures S4D, S4E, and 3B). We found significant enrichment of functional similarity within the 60-gene set compared to background genes ($p < 2.6e{-}16$; Figure 3B). Strikingly, we also found a significant enrichment between the 60 genes and a set of known PD genes (Figure 3B).

Within the set of 60 DE genes, those downregulated early in the proposed case-control axis include genes implicated in neuronal function (γ-synuclein [*SNCG*], brain-derived neurotrophic factor [*BDNF*], and dopamine receptor D2 [*DRD2*]); genes involved in microtubule-associated protein tau (*MAPT*) splicing, microtubule function and formation, and neurite and axonal outgrowth; genes involved in protein secretion and trafficking; and protein kinase C (PKC) pathway genes. Genes identified as upregulated late in the process include the ER stress genes protein disulfide isomerase family member 6 (*PDIA6*), FK506 binding protein 9 (*FKBP9*), and ER oxidoreductase 1 alpha (*ERO1A*). The upregulation of ER stress genes is consistent with our previous findings, in which ER stress was increased in

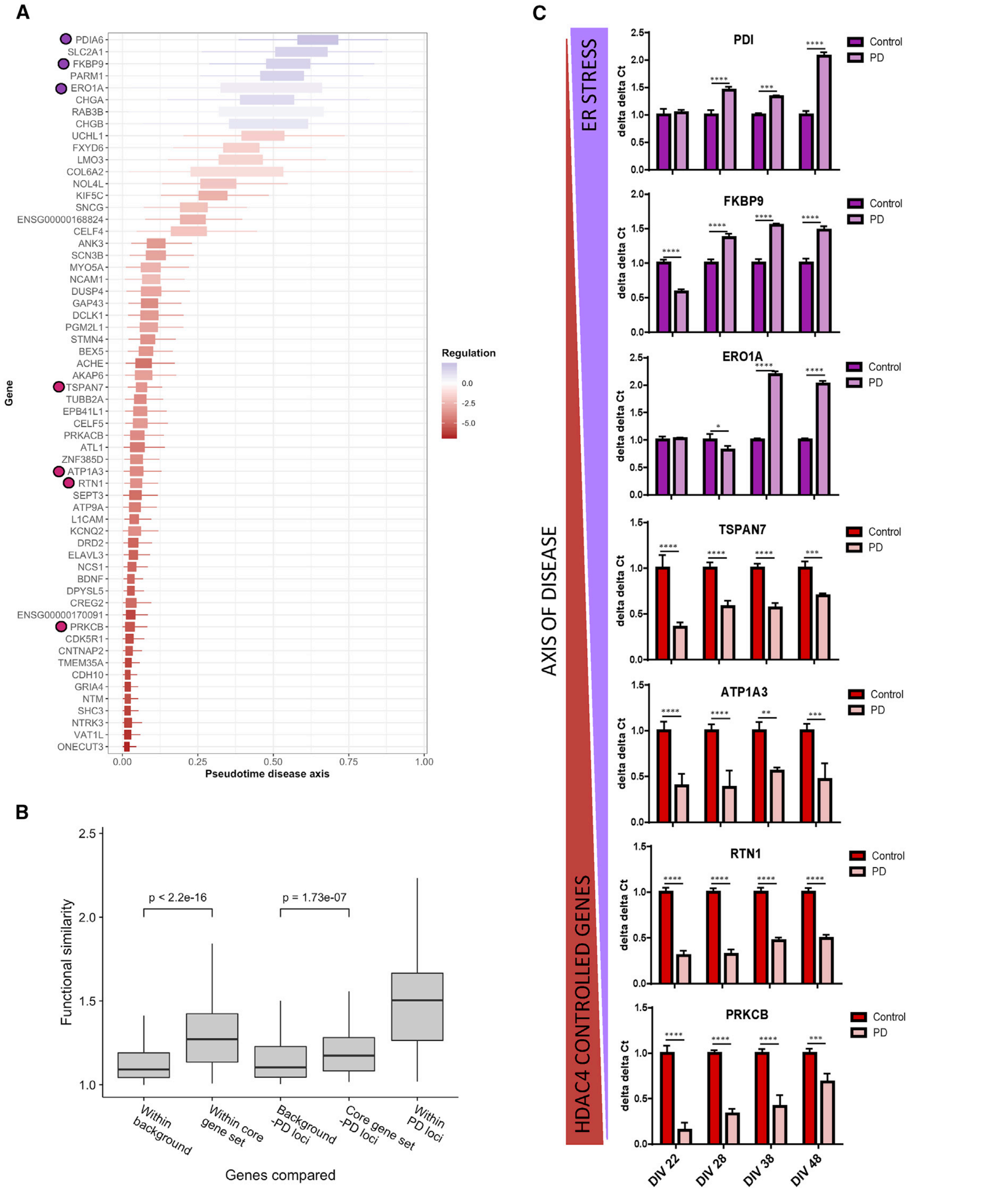

Figure 3. Pseudotime Analysis Temporally Orders the Core Set of 60 Functionally Similar Genes DE in Both the Bulk and Single-Cell RNA-Seq between Control and PD *GBA-N370S* Patients

(A) Refined transcriptomic disease axis analysis of the core gene set of 60 genes. The control-disease single-cell transcriptomic axis was re-inferred with the 60 genes alone using a parametric factor analysis model that associated each gene with a point along the axis at which it was upregulated or downregulated.

(B) The phenotypic linkage network demonstrates a higher functional similarity of the 60-gene set with each other compared to a background control set ($p < 2.2e{-}16$). This higher functional similarity was also identified between the 60-gene set and a group of known PD loci, compared to a background control set ($p = 8.52e{-}08$). The high functional similarity of PD genes to each other is used as a positive control.

(C) Along the axis of disease, the downregulation of HDAC4-controlled genes (*PRKCB*, *RTN1*, *ATP1A3*, and *TSPAN7*) at 22 DIV precedes the upregulation of ER stress genes (*ERO1A*, *FKBP9*, and *PDI*) at 38 DIV. Data are represented as mean ± SD (*p < 0.05, **p < 0.01, ***p < 0.001, and ****p < 0.0001). The locations of the HDAC4 and ER genes analyzed from the core 60 set are marked on (A).

iPSC-derived dopamine neurons from PD *GBA-N370S* patients (Fernandes et al., 2016).

We further refined the single-cell transcriptomic axis of 60 genes using a recent Bayesian approach that learns transcriptomic trajectories directly from pre-specified genes using single-cell expression data. Based on nonlinear factor analysis, this approach models a small gene set in terms of "switch-like" upregulation or downregulation along the latent (pseudotime) axis, jointly inferring the pseudotimes along with all model parameters. Crucially, it probabilistically assigns a position along the axis associated with the upregulation or downregulation of each of the 60 genes, and we can anchor the direction of the axis as proceeding from those GBA1 and GBA2 iPSC-derived dopamine neurons that are most similar to controls. We hypothesize that this axis represents the continuous progression of these cells through a modeled disease-relevant process, moving from a more control-like state to a more PD-relevant disease state, and where the order of gene regulatory variation along this axis reflects this modeled disease process (Figure 3A).

Analysis of the core set of 60 DE genes using ingenuity pathway analysis (IPA) (QIAGEN) identified histone deacetylase 4 (HDAC4) as a repressor of a set of genes downregulated early in the pseudotemporal profile (Figure S5A). Although total levels of HDAC4 protein were unchanged between controls and PD *GBA-N370S* patients (Figure S5B), the downregulation of four of the HDAC4-regulated genes (*TSPAN7*, *ATP1A3*, *RTN1*, and *PRKCB*) in PD *GBA-N370S* patient-derived neurons was experimentally confirmed (Figure S5C).

We next sought to validate the proposed temporal order of gene expression events in the development of disease pathophysiology in PD *GBA-N370S* neurons. qRT-PCR analysis of *TSPAN7*, *ATP1A3*, *RTN1*, and *PRKCB* confirmed that these four "early" genes, predicted to be downregulated by HDAC4, are repressed early in the differentiation at 22 DIV (days *in vitro*). The "late" genes (*ERO1A*, *FKBP9*, and *PDIA6*), predicted to be upregulated as part of a subsequent ER stress response, typically increase in expression post-22 DIV, with all three increased at 38 and 48 DIV (Figure 3C).

HDAC4 Is Mislocalized to the Nucleus and Participates in the Repression of Gene Expression in PD *GBA-N370S* iPSC-Derived Dopamine Neurons

HDAC4, a class IIa histone deacetylase, shuttles between the cytoplasm and the nucleus, where it acts as a transcriptional repressor. We observed an increase in nuclear localization of HDAC4 in PD *GBA-N370S* iPSC-derived dopamine neurons compared to controls at DIV 45, consistent with the downregulation of HDAC4 controlled genes within our set of 60 genes (Figure 4A). This HDAC4 nuclear mislocalization was not observed in iPSC-derived non-dopaminergic neurons of PD *GBA-N370S* patients (Figure S6).

Modulating HDAC4 Localization or Activity Corrects the Downregulation of HDAC4-Repressed Genes and Ameliorates ER Stress Phenotypes

To examine the effect of HDAC4 repression on the set of downregulated genes, we used four modulators of HDAC4 activity or localization, currently in clinical use for unrelated conditions. Tasquinimod is an allosteric inhibitor of the association of HDAC4 with the nuclear N-Cor/HDAC3-associated repressor complex (Isaacs et al., 2013), and okadaic acid (OA), cantharidin, and LB-100 (LB-100) all inhibit protein phosphatase 2 (PP2A)-mediated dephosphorylation of HDAC4, which reduces its nuclear localization (Gordon et al., 2015; Paroni et al., 2008; Pei et al., 2016).

Treatment of PD *GBA-N370S* iPSC-derived dopamine neurons with each of the three PP2A inhibitor compounds reduced the nuclear localization of HDAC4, correcting HDAC4 mislocalization in *GBA-N370S* dopamine neurons to that of controls (Figure 4B). The addition of the HDAC4 allosteric inhibitor tasquinimod did not reduce the HDAC4 nuclear localization (Figure 4B) consistent with its mode of action, which does not involve HDAC4 relocalization. We next examined the impact on gene expression phenotypes of treating iPSC-derived dopamine neurons with the HDAC4 modulators. Treatment with all four compounds corrected, or even reversed, the reduction in expression of all four HDAC4-controlled genes reduced early in the pseudotemporal axis (*PRKCB*, *RTN1*, *ATP1A3*, and *TSPAN7*) in PD *GBA-N370S* iPSC-derived dopamine neurons at DIV 45 (Figure 5). Furthermore, compounds ameliorated the increase seen in the three ER stress genes (*ERO1A*, *FKBP9*, and *PDI*), late in the pseudotemporal axis, at the RNA and protein level (Figures 5 and S7A).

HDAC4 Modulation Corrects Perturbations in the Autophagy and Lysosomal Pathway in PD *GBA-N370S* iPSC-Derived Dopamine Neurons

In addition to increased ER stress, we have previously observed perturbations in the autophagic and lysosomal pathway and increased release of α-synuclein in PD *GBA-N370S* iPSC-derived dopamine neurons (Fernandes et al., 2016). Treating PD *GBA-N370S* iPSC-derived dopamine neurons with the HDAC4 allosteric inhibitor tasquinimod or the representative PP2A inhibitor cantharidin corrected the increase in autophagosome number assessed by LC3-II levels (Figure 6A) through decreased autophagic induction rather than increasing flux (Figures S7B–S7D), reduced the increase in lysosomal accumulation measured by LAMP1 (Figures 6B and 6C), increased lysosomal activity (Figure 6D), and reduced the increased release of α-synuclein into the extracellular medium (Figure 6E).

Nuclear Mislocalization of HDAC4 and Related Perturbations in Gene Expression Are Observed in Idiopathic PD Cases

To address whether HDAC4 mislocalization is a disease mechanism relevant to PD beyond carriers of *GBA* mutations, we examined HDAC4 mislocalization and perturbation of gene expression in dopamine neurons differentiated from iPSC lines generated from four idiopathic PD cases and three age-matched controls. An increase in HDAC4 nuclear localization was observed in iPSC-derived dopamine neurons from two of the four idiopathic PD cases (Figures 7A and 7B). Furthermore, the reduction of expression of the HDAC4-regulated genes *TSPAN7*, *ATP1A3*, *RTN1*, and *PRKCB*, and the upregulation of the ER stress genes *ERO1A*, *PDIA6*, and *FKBP9*, was observed in iPSC-derived dopamine neurons from the same two idiopathic PD cases, which exhibited HDAC4 mislocalization (Figures 7C and 7D).

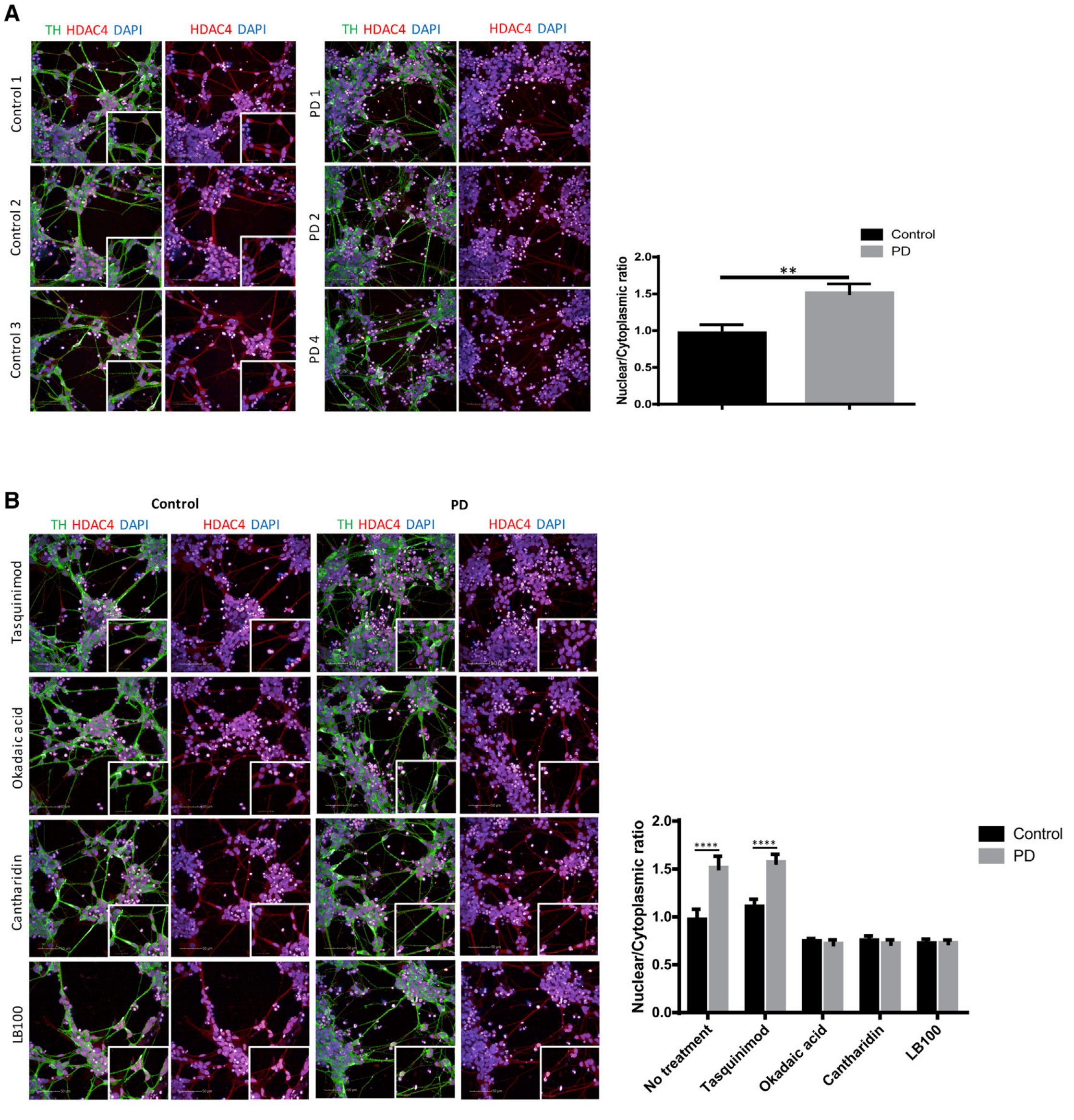

Figure 4. Modulation of PP2A Activity Corrects HDAC4 Nuclear Mislocalization in PD *GBA-N370S* iPSC-Derived Dopamine Neurons

(A) Cytoplasmic and nuclear localization of HDAC4 in control and PD *GBA-N370S* dopamine neurons shown by immunofluorescence at 45 DIV—TH, green; HDAC4, red; DAPI, blue; HDAC4/DAPI nuclear colocalization, purple. The HDAC4 nuclear/cytoplasmic ratio is significantly increased in PD *GBA-N370S* patients. Data are represented as mean ± SD (**p < 0.01).

(B) HDAC4 cellular localization in the presence or absence of tasquinimod (HDAC4 allosteric inhibitor) or okadaic acid, cantharidin, and LB-100 (PP2A inhibitors) at 45 DIV—TH, green; HDAC, red; DAPI, blue; and HDAC4/DAPI nuclear colocalization, purple. The three PP2A inhibitors correct HDAC4 nuclear mislocalization in PD *GBA-N370S* patient-derived dopamine neurons compared to no treatment. In contrast, tasquinimod, a HDAC4 allosteric inhibitor, has no effect on HDAC4 localization. Data are represented as mean ± SD (****p < 0.0001).

DISCUSSION

Applying cell type purification and a combination of bulk and single-cell gene expression profiling to iPSC-derived dopamine neurons from three *GBA-N370S* patients, our study identified disease-distinguishing molecular etiologies and revealed a temporal ordering of gene expression variation that proposed a role for the transcriptional regulator HDAC4 in upstream variation. The pharmacological modulation of HDAC4 activity or localization confirmed this finding by the rescue of downstream expression variation and correction of cellular phenotypes previously shown in this model of PD.

Our FACS-based purification method is well-suited to deep single-cell profiling. Although the cell fixation necessary for sorting creates a 3′ bias in transcript coverage, our gene level coverage was high, enabling subsequent studies. Cells can be clustered post-sequencing according to their expression profiles, but the cellular heterogeneity in these cultures would

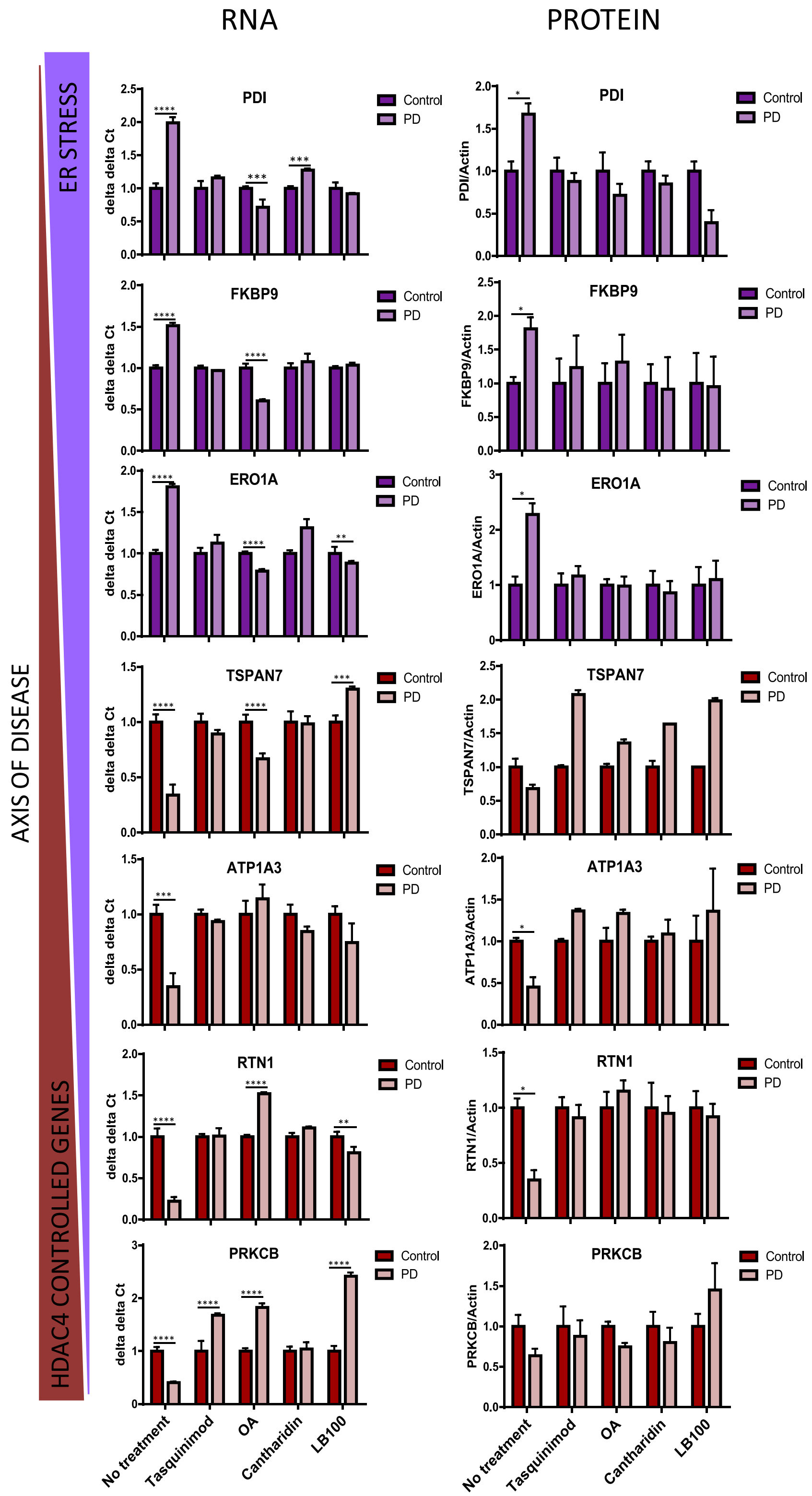

Figure 5. Modulation of HDAC4 Activity or Localization Corrects the Downregulation of HDAC4-Controlled Genes in PD *GBA-N370S* iPSC-Derived Dopamine Neuron Cultures and Ameliorates PD *GBA-N370S* ER Stress Phenotypes

Expression of four HDAC4-regulated genes (*TSPAN7*, *ATP1A3*, *RTN1*, and *PRKCB*; bottom) and three ER stress genes (*PDIA6*, *FKBP9*, and *ERO1A*; top) at the RNA (left) and protein (right) levels in the presence and absence of HDAC4-modifying drugs tasquinimod, okadaic acid, cantharidin, and LB-100 in PD *GBA-N370S* and control patient-derived neurons at 45 DIV. The upregulation of HDAC4-repressed genes in PD *GBA-N370S* iPSC-derived dopamine neurons by all four compounds was accompanied by a decrease in ER stress. Data are represented as mean ± SEM (*p < 0.05, **p < 0.01, ***p < 0.001, and ****p < 0.0001).

have halved our capture of dopaminergic neurons. Our robust pseudotemporal analyses require only ~150 single-cell transcriptomic profiles to reveal disease-relevant perturbations.

Beyond cell-type heterogeneity, our study exploited significant intra-culture heterogeneity to unpick the disease processes being modeled. First, we identified a distinct molecular perturbation present in iPSC-derived dopamine neurons generated from *GBA-N370S* patient 3 (GBA3). Despite an initial diagnosis of PD and possessing a genetic variant strongly associated with PD, this patient's cellular profile prompted a clinical reassessment, leading to the revised diagnosis of PSP. Although superficially similar in clinical presentation, PSP is a tauopathy with a cellular pathophysiology distinct to the α-synucleinopathy PD. Each of this patient's single-cell profiles proved an effective technical replicate in the analyses. Profiling this patient's cells revealed a distinct perturbation, which was further validated in two additional iPSC-derived dopamine neuron lines from the same patient.

A second source of cellular heterogeneity is the varying progression of each cell through the same disease process over time. Although a bulk expression profile averages across the cellular population, obscuring variation, a single-cell approach is able to exploit this heterogeneity and reveals insights into dynamic processes across a pseudotemporal axis. The ability to infer the temporal nature of disease progression allowed us to explore the relationship between early biological changes in gene expression and their influence on later disease phenotypes.

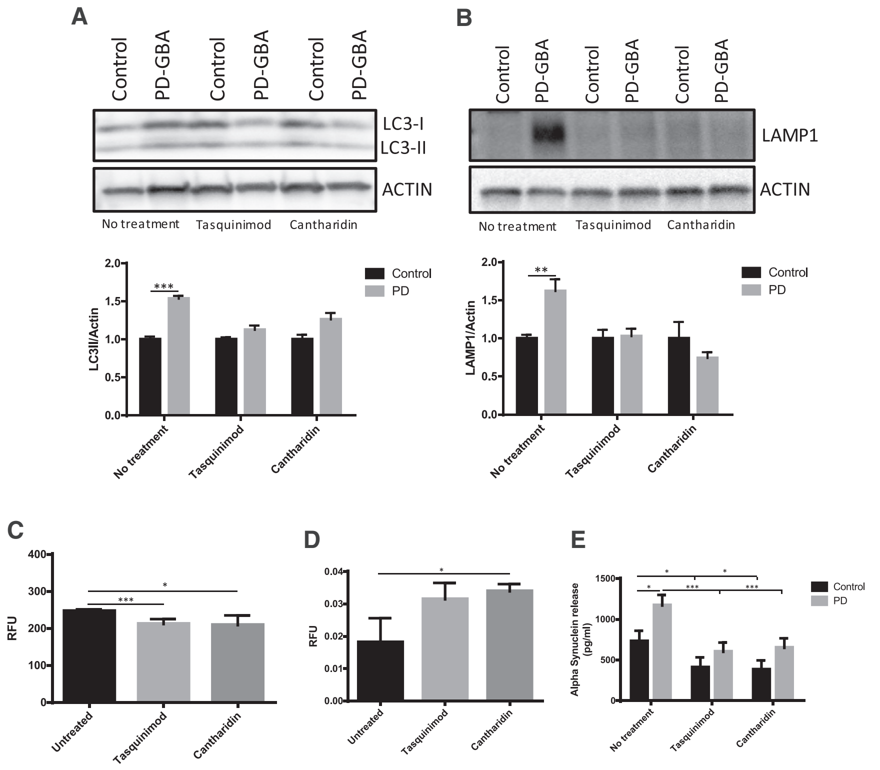

Figure 6. Modulation of HDAC4 Activity or Localization Rescues Deficits in the Autophagic and Lysosomal Pathway and Reduces α-Synuclein Release in PD *GBA-N370S* iPSC-Derived Dopamine Neurons

(A and B) Modulation of HDAC4 activity by allosteric inhibition of HDAC4 (tasquinimod) or inhibition of PP2A (cantharidin) rescues the increase in autophagosomal (LC3-II; A) and lysosomal (LAMP1; B) compartments seen by western blot in PD *GBA-N370S* patient iPSC-derived neurons compared to controls.

(C) The reduction of lysosomes in PD *GBA-N370S* iPSC-derived dopamine neurons treated with tasquinimod or cantharidin was confirmed by a decrease in lysosome punctae by immunofluorescence.

(D) Modulation of HDAC4 increases lysosomal activity in PD *GBA-N370S* iPSC-derived neurons measured by DQ-BSA cleavage.

(E) Tasquinimod or cantharidin reduces the increase in α-synuclein release seen in PD *GBA-N370S* patient-derived neurons compared to controls.

Data are represented as mean ± SEM (*p < 0.05, **p < 0.01, and ***p < 0.001).

We identified HDAC4 as a master regulator of a number of genes downregulated early in the disease axis. Unlike class I HDACs, which reside permanently in the nucleus, HDAC4 acts as part of the HDAC4/N-CoR/HDAC3 complex that shuttles between the cytoplasm and the nucleus, repressing the expression of genes important in synaptic function and neuronal health. Under normal conditions, phosphorylated HDAC4 is retained in the cytoplasm, but upon dephosphorylation of the Ser298 residue by the catalytic subunit of PP2A, HDAC4 relocalizes to the nucleus. Although HDAC4 was not DE in PD *GBA-N370S* patient-derived dopamine neurons compared to controls, an increase in the nuclear-to-cytoplasmic ratio of HDAC4 was identified, consistent with the downregulation of DE genes in the core set under the transcriptional control of HDAC4.

We therefore hypothesize that downregulation of HDAC4-controlled genes due to the mislocalization of HDAC4 in the nucleus *early* in the disease may contribute to driving ER stress *later* in neurodegeneration. For example, mutations in the gene *ATP1A3*, which is downregulated by HDAC4, cause a rare rapid-onset dystonia-parkinsonism and is linked to altering intracellular calcium levels, which could impact on the ER, the principal intracellular store of calcium (Blanco-Arias et al., 2009). Similarly, *PRKCB* participates at mitochondrial-ER-associated membrane (MAM) sites, playing a crucial role in the phosphorylation of the p66^{Shc} protein, which is involved in the regulation of calcium homeostasis between these two organelles (Pinton and Rizzuto, 2008).

Deficits in calcium signaling may also cause the increased nuclear localization of HDAC4 in PD *GBA-N370S* patient-derived dopamine neurons. HDAC4 is known to regulate genes involved in synaptic activity and memory and neuronal health (Sando et al., 2012). As cytoplasmic retention of class IIa HDACs requires calcium-dependent phosphorylation through calcium and/or calmodulin-dependent kinases, elevated cytoplasmic calcium caused by influx through voltage-gated ion channels in highly active neurons maintains HDAC4 cytoplasmic retention. Conversely, loss of synaptic excitation due to neurodegeneration may contribute to HDAC4 nuclear localization and repression of genes that promote neuronal survival. PD *GBA-N370S* patient-derived dopamine neurons are known to exhibit impaired cellular calcium homeostasis (Schöndorf et al., 2014), and low synaptic calcium levels in hippocampal and cerebellar granule cell cultures triggered the shuttling of HDAC4 from dendritic spines to the nucleus (Bolger and Yao, 2005; Chawla et al., 2003).

Pharmacological modulation of HDAC4 activity or localization corrected cellular phenotypes previously described in PD *GBA-N370S* patient-derived dopamine neurons, alleviating ER stress to reduce autophagic induction, suggesting HDAC4 as a therapeutic target for PD. All compounds tested are currently in clinical development for unrelated conditions, principally cancer. Decreased HDAC4 nuclear localization through increased phosphorylation and cytoplasmic retention was achieved through inhibition of PP2A. PP2A dephosphorylates multiple targets in addition to HDAC4, including the major neurodegenerative proteins tau and α-synuclein, which may prevent prolonged clinical use. More interesting is the use of the allosteric inhibitor tasquinimod to inhibit formation of the HDAC4/N-CoR/HDAC3 repression complex by locking HDAC4 in an inactive form (Isaacs et al., 2013). Tasquinimod has been tested through phase II and III clinical trials to treat prostate cancer with a good safety profile (Armstrong et al., 2013; Sternberg et al., 2016). The compound

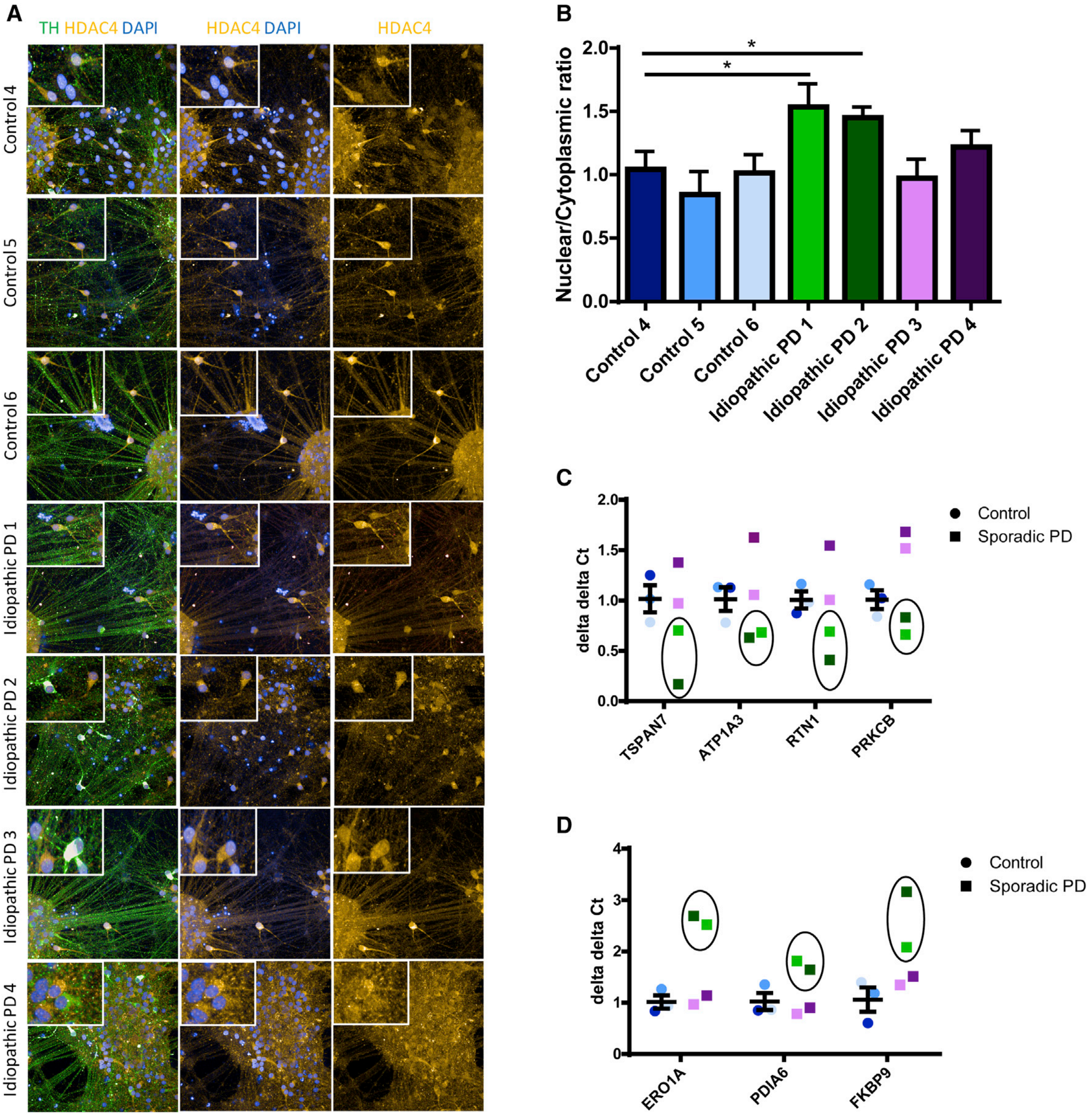

Figure 7. Nuclear Mislocalization of HDAC4 and Related Perturbations in Gene Expression Are Observed in Idiopathic PD Cases

(A) Cytoplasmic and nuclear localization of HDAC4 in control and idiopathic PD iPSC-derived dopamine neurons shown by immunofluorescence at 45 DIV—TH, green; HDAC4, yellow; DAPI, blue.

(B) The HDAC4 nuclear/cytoplasmic ratio is significantly increased in two of the four idiopathic PD patients. Data are represented as mean ± SEM (*$p < 0.05$).

(C and D) A (C) decrease in the expression of HDAC4-controlled genes: *TSPAN7*; *ATP1A3*; *RTN1*; and *PRKCBI* and an (D) increase in the expression of ER stress genes: *ERO1A*; *PDIA6*; and *FKBP9* is observed in the same two idiopathic PD cases that display HDAC4 mislocalization.

is well-tolerated in patients for up to 3 or 4 years with few dose interruptions or reductions. HDAC4 is considered to be a potential therapeutic target in Huntington's disease (HD), as a heterozygous *Hdac4*$^{+/-}$ background rescued neuronal function in a HD mouse model (Mielcarek et al., 2013) but has yet to be explored therapeutically in PD.

To investigate whether these disease mechanisms are relevant to PD beyond carriers of *GBA* mutations, we extended our study to include iPSC-derived dopamine neurons from idiopathic PD cases. Remarkably, we found that increased HDAC4 nuclear localization was seen in iPSC-derived dopamine neurons from two of four idiopathic PD cases. Furthermore, the same perturbation of expression, being the downregulation of HDAC4-regulated genes *TSPAN7*, *ATP1A3*, *RTN1*, and *PRKCB* and the upregulation of ER stress genes *ERO1A*, *PDIA6*, and *FKBP9*, was seen in the same two idiopathic PD cases exhibiting HDAC4 mislocalization. These data show that findings from *GBA* PD extrapolate to a subset of idiopathic PD cases. Heterogeneity between idiopathic patients is expected in a disease with complex polygenic inheritance, leading to a variable level of cell-autonomous effects in different individuals, and one might expect the genetic contribution to be greater in some idiopathic

patients than others. Our findings are consistent with recent studies (Hsieh et al., 2016; Sánchez-Danés et al., 2012; Nenasheva et al., 2017; Tolosa et al., 2018; George et al., 2018), which have found cellular phenotypes or transcriptomic perturbations in iPSC-derived dopamine neurons from idiopathic PD patients.

Overall, our work applied high-resolution single-cell analysis to iPSC-based disease models, exploiting the cellular heterogeneity present even within a purified single cell type, in this case, iPSC-derived dopamine neurons from PD patients. We have shown the disease process to be a dynamic event and identified HDAC4 as a key regulator of the *early* molecular changes that lead to *late* pathological processes. Our approach is applicable to other diseases as a means to uncover disease mechanisms and discover potential therapeutic targets.

STAR★METHODS

Detailed methods are provided in the online version of this paper and include the following:

- KEY RESOURCES TABLE
- CONTACT FOR REAGENT AND RESOURCE SHARING
- EXPERIMENTAL MODEL AND SUBJECT DETAILS
  - iPSC lines and participation recruitment
  - Subject details
  - Culture, reprogramming and characterization of primary fibroblasts
  - Generation and characterization of iPSC derived dopamine neurons
- METHOD DETAILS
  - Purification of iPSC dopaminergic neurons by flow cytometry
  - RNA preparation of bulk RNA-seq samples
  - Smart-seq2, RNA library construction and sequencing
  - RNA-seq read alignment and expression quantification
  - Quality control of single-cell RNA-seq
  - Differential expression analysis
  - Single-cell pseudotime analysis
  - Identification of pathway activation in GBA 3
  - Phenotypic linkage network construction
  - qRT-PCR, immunocytochemistry and western blot
  - DQ-BSA
  - α-synuclein release
- QUANTIFICATION AND STATISTICAL ANALYSIS

SUPPLEMENTAL INFORMATION

Supplemental Information includes seven figures and two tables and can be found with this article online at https://doi.org/10.1016/j.stem.2018.10.023.

ACKNOWLEDGMENTS

The work was supported by the Monument Trust Discovery Award from Parkinson's UK. C.W. was supported by the Medical Research Council, UK. The Oxford Martin School (LC0910-004) and the Wellcome Trust (WTISSF121302) provide core support to the James Martin Stem Cell Facility within the Sir William Dunn School of Pathology (S.A.C.). The OPDC Discovery cohort was supported by the National Institute for Health Research (NIHR) Oxford Biomedical Research Centre based at Oxford University Hospitals NHS Trust and University of Oxford and the Dementia and Neurodegenerative Diseases Research Network (DeNDRoN). Single-cell transcriptomics at the Oxford Genomics Centre was supported by a Wellcome Trust core grant to the Wellcome Centre for Human Genetics, reference 090532/Z/09/Z. We also thank Christine Klein (Lübeck) for clinical expertise in PD and Uroosa Chughtai (Oxford) for valuable technical assistance in cell culture. The research leading to these results has received support from the Innovative Medicines Initiative Joint Undertaking (IMIJU) under grant agreement n_115439, resources of which are composed of financial contribution from the European Union's Seventh Framework Programme (FP7/2007-2013) and EFPIA companies' in kind contribution. This publication reflects only the author's views and neither the IMI JU nor EFPIA nor the European Commission are liable for any use that may be made of the information contained therein. Funding to pay the Open Access publication charges for this article was provided by Parkinson's UK (COAF) (J-1403) and Wellcome Trust (092762/Z/10/Z).

AUTHOR CONTRIBUTIONS

C.L. differentiated iPSC lines and generated experimental data; K.R.C. and C.W. undertook bioinformatics analysis; B.J.R. performed IPA analysis and generated experimental data; P.C. generated experimental data; M.A. and R.B. performed the RNA-seq; J.V., O.V.P., and S.A.C. generated and banked the iPSC lines; and M.T.H., F.B., and M.K. provided clinical patient assessment. R.W.-M. supervised the experimental work; R.W.-M. and C.W. devised the study.

DECLARATION OF INTERESTS

The authors declare no competing interests.

Received: March 5, 2018
Revised: July 13, 2018
Accepted: October 23, 2018
Published: November 29, 2018

SUPPORTING CITATIONS

The following reference appears in the Supplemental Information: Takahashi et al. (2007).

REFERENCES

Armstrong, A.J., Häggman, M., Stadler, W.M., Gingrich, J.R., Assikis, V., Polikoff, J., Damber, J.E., Belkoff, L., Nordle, Ö., Forsberg, G., et al. (2013). Long-term survival and biomarker correlates of tasquinimod efficacy in a multicenter randomized study of men with minimally symptomatic metastatic castration-resistant prostate cancer. Clin. Cancer Res. *19*, 6891–6901.

Baker, M.G., and Graham, L. (2004). The journey: Parkinson's disease. BMJ *329*, 611–614.

Beavan, M.S., and Schapira, A.H. (2013). Glucocerebrosidase mutations and the pathogenesis of Parkinson disease. Ann. Med. *45*, 511–521.

Beevers, J.E., Lai, M.C., Collins, E., Booth, H.D.E., Zambon, F., Parkkinen, L., Vowles, J., Cowley, S.A., Wade-Martins, R., and Caffrey, T.M. (2017). MAPT genetic variation and neuronal maturity alter isoform expression affecting axonal transport in iPSC-derived dopamine neurons. Stem Cell Reports *9*, 587–599.

Blanco-Arias, P., Einholm, A.P., Mamsa, H., Concheiro, C., Gutiérrez-de-Terán, H., Romero, J., Toustrup-Jensen, M.S., Carracedo, A., Jen, J.C., Vilsen, B., and Sobrido, M.J. (2009). A C-terminal mutation of ATP1A3 underscores the crucial role of sodium affinity in the pathophysiology of rapid-onset dystonia-parkinsonism. Hum. Mol. Genet. *18*, 2370–2377.

Bolger, T.A., and Yao, T.P. (2005). Intracellular trafficking of histone deacetylase 4 regulates neuronal cell death. J. Neurosci. *25*, 9544–9553.

Bray, N.L., Pimentel, H., Melsted, P., and Pachter, L. (2016). Near-optimal probabilistic RNA-seq quantification. Nat. Biotechnol. *34*, 525–527.

Brennecke, P., Anders, S., Kim, J.K., Kołodziejczyk, A.A., Zhang, X., Proserpio, V., Baying, B., Benes, V., Teichmann, S.A., Marioni, J.C., and Heisler, M.G. (2013). Accounting for technical noise in single-cell RNA-seq experiments. Nat. Methods *10*, 1093–1095.

Campbell, K.R., and Yau, C. (2017). switchde: inference of switch-like differential expression along single-cell trajectories. Bioinformatics *33*, 1241–1242.

Chawla, S., Vanhoutte, P., Arnold, F.J., Huang, C.L., and Bading, H. (2003). Neuronal activity-dependent nucleocytoplasmic shuttling of HDAC4 and HDAC5. J. Neurochem. *85*, 151–159.

Du, K., Liu, M.Y., Zhong, X., and Wei, M.J. (2017). Decreased circulating Zinc levels in Parkinson's disease: a meta-analysis study. Sci. Rep. *7*, 3902.

Fearnley, J.M., and Lees, A.J. (1991). Ageing and Parkinson's disease: substantia nigra regional selectivity. Brain *114*, 2283–2301.

Fernandes, H.J., Hartfield, E.M., Christian, H.C., Emmanoulidou, E., Zheng, Y., Booth, H., Bogetofte, H., Lang, C., Ryan, B.J., Sardi, S.P., et al. (2016). ER stress and autophagic perturbations lead to elevated extracellular α-synuclein in GBA-N370S Parkinson's iPSC-derived dopamine neurons. Stem Cell Reports *6*, 342–356.

Forsleff, L., Schauss, A.G., Bier, I.D., and Stuart, S. (1999). Evidence of functional zinc deficiency in Parkinson's disease. J. Altern. Complement. Med. *5*, 57–64.

George, G., Singh, S., Lokappa, S.B., and Varkey, J. (2018). Gene co-expression network analysis for identifying genetic markers in Parkinson's disease - a three-way comparative approach. Genomics *7543*, 30282–30289.

Gonera, E.G., van't Hof, M., Berger, H.J., van Weel, C., and Horstink, M.W. (1997). Symptoms and duration of the prodromal phase in Parkinson's disease. Mov. Disord. *12*, 871–876.

Gordon, I.K., Lu, J., Graves, C.A., Huntoon, K., Frerich, J.M., Hanson, R.H., Wang, X., Hong, C.S., Ho, W., Feldman, M.J., et al. (2015). Protein phosphatase 2A inhibition with LB100 enhances radiation-induced mitotic catastrophe and tumor growth delay in glioblastoma. Mol. Cancer Ther. *14*, 1540–1547.

Haenseler, W., Zambon, F., Lee, H., Vowles, J., Rinaldi, F., Duggal, G., Houlden, H., Gwinn, K., Wray, S., Luk, K.C., et al. (2017). Excess α-synuclein compromises phagocytosis in iPSC-derived macrophages. Sci. Rep. *7*, 9003.

Haghverdi, L., Büttner, M., Wolf, F.A., Buettner, F., and Theis, F.J. (2016). Diffusion pseudotime robustly reconstructs lineage branching. Nat. Methods *13*, 845–848.

Honti, F., Meader, S., and Webber, C. (2014). Unbiased functional clustering of gene variants with a phenotypic-linkage network. PLoS Comput. Biol. *10*, e1003815.

Hruska, K.S., LaMarca, M.E., Scott, C.R., and Sidransky, E. (2008). Gaucher disease: mutation and polymorphism spectrum in the glucocerebrosidase gene (GBA). Hum. Mutat. *29*, 567–583.

Hsieh, C.H., Shaltouki, A., Gonzalez, A.E., Bettencourt da Cruz, A., Burbulla, L.F., St Lawrence, E., Schüle, B., Krainc, D., Palmer, T.D., and Wang, X. (2016). Functional impairment in miro degradation and mitophagy is a shared feature in familial and sporadic parkinson's disease. Cell Stem Cell *19*, 709–724.

Hughes, A.J., Daniel, S.E., Kilford, L., and Lees, A.J. (1992). Accuracy of clinical diagnosis of idiopathic Parkinson's disease: a clinico-pathological study of 100 cases. J. Neurol. Neurosurg. Psychiatry *55*, 181–184.

Ilicic, T., Kim, J.K., Kolodziejczyk, A.A., Bagger, F.O., McCarthy, D.J., Marioni, J.C., and Teichmann, S.A. (2016). Classification of low quality cells from single-cell RNA-seq data. Genome Biol. *17*, 29.

Isaacs, J.T., Antony, L., Dalrymple, S.L., Brennen, W.N., Gerber, S., Hammers, H., Wissing, M., Kachhap, S., Luo, J., Xing, L., et al. (2013). Tasquinimod is an allosteric modulator of HDAC4 survival signaling within the compromised cancer microenvironment. Cancer Res. *73*, 1386–1399.

Ji, Z., and Ji, H. (2016). TSCAN: pseudo-time reconstruction and evaluation in single-cell RNA-seq analysis. Nucleic Acids Res. *44*, e117.

Kasten, M., Hagenah, J., Graf, J., Lorwin, A., Vollstedt, E.-J., Peters, E., Katalinic, A., Raspe, H., and Klein, C. (2013). Cohort profile: a population-based cohort to study non-motor symptoms in parkinsonism (EPIPARK). Int. J. Epidemiol. *42*, 128–128k.

Kim, D., Langmead, B., and Salzberg, S.L. (2015). HISAT: a fast spliced aligner with low memory requirements. Nat. Methods *12*, 357–360.

Kiselev, V.Y., Kirschner, K., Schaub, M.T., Andrews, T., Yiu, A., Chandra, T., Natarajan, K.N., Reik, W., Barahona, M., Green, A.R., and Hemberg, M. (2017). SC3: consensus clustering of single-cell RNA-seq data. Nat. Methods *14*, 483–486.

Kriks, S., Shim, J.W., Piao, J., Ganat, Y.M., Wakeman, D.R., Xie, Z., Carrillo-Reid, L., Auyeung, G., Antonacci, C., Buch, A., et al. (2011). Dopamine neurons derived from human ES cells efficiently engraft in animal models of Parkinson's disease. Nature *480*, 547–551.

Krueger, F. (2015). Trim Galore. A wrapper tool around Cutadapt and FastQC to consistently apply quality and adapter trimming to FastQ files. Babraham Bioinformatics, https://www.bioinformatics.babraham.ac.uk/projects/trim_galore/.

Lamble, S., Batty, E., Attar, M., Buck, D., Bowden, R., Lunter, G., Crook, D., El-Fahmawi, B., and Piazza, P. (2013). Improved workflows for high throughput library preparation using the transposome-based Nextera system. BMC Biotechnol. *13*, 104.

Love, M.I., Huber, W., and Anders, S. (2014). Moderated estimation of fold change and dispersion for RNA-seq data with DESeq2. Genome Biol. *15*, 550.

Lun, A.T., McCarthy, D.J., and Marioni, J.C. (2016). A step-by-step workflow for low-level analysis of single-cell RNA-seq data with Bioconductor. F1000Res. *5*, 2122.

McCarthy, D.J., Campbell, K.R., Lun, A.T., and Wills, Q.F. (2017). Scater: pre-processing, quality control, normalization and visualization of single-cell RNA-seq data in R. Bioinformatics *33*, 1179–1186.

Mielcarek, M., Landles, C., Weiss, A., Bradaia, A., Seredenina, T., Inuabasi, L., Osborne, G.F., Wadel, K., Touller, C., Butler, R., et al. (2013). HDAC4 reduction: a novel therapeutic strategy to target cytoplasmic huntingtin and ameliorate neurodegeneration. PLoS Biol. *11*, e1001717.

Nenasheva, V.V., Novosadova, E.V., Makarova, I.V., Lebedeva, O.S., Grefenshtein, M.A., Arsenyeva, E.L., Antonov, S.A., Grivennikov, I.A., and Tarantul, V.Z. (2017). The transcriptional changes of trim genes associated with parkinson's disease on a model of human induced pluripotent stem cells. Mol. Neurobiol. *54*, 7204–7211.

Park, J.S., Koentjoro, B., Veivers, D., Mackay-Sim, A., and Sue, C.M. (2014). Parkinson's disease-associated human ATP13A2 (PARK9) deficiency causes zinc dyshomeostasis and mitochondrial dysfunction. Hum. Mol. Genet. *23*, 2802–2815.

Paroni, G., Cernotta, N., Dello Russo, C., Gallinari, P., Pallaoro, M., Foti, C., Talamo, F., Orsatti, L., Steinkühler, C., and Brancolini, C. (2008). PP2A regulates HDAC4 nuclear import. Mol. Biol. Cell *19*, 655–667.

Pei, Y., Liu, K.W., Wang, J., Garancher, A., Tao, R., Esparza, L.A., Maier, D.L., Udaka, Y.T., Murad, N., Morrissy, S., et al. (2016). HDAC and PI3K antagonists cooperate to inhibit growth of MYC-driven medulloblastoma. Cancer Cell *29*, 311–323.

Picard Toolkit (2018). Picard Toolkit. Broad Institute, http://broadinstitute.github.io/picard/.

Picelli, S., Björklund, A.K., Faridani, O.R., Sagasser, S., Winberg, G., and Sandberg, R. (2013). Smart-seq2 for sensitive full-length transcriptome profiling in single cells. Nat. Methods *10*, 1096–1098.

Pierson, E., and Yau, C. (2015). ZIFA: dimensionality reduction for zero-inflated single-cell gene expression analysis. Genome Biol. *16*, 241.

Pinton, P., and Rizzuto, R. (2008). p66Shc, oxidative stress and aging: importing a lifespan determinant into mitochondria. Cell Cycle *7*, 304–308.

R Development Core Team (2008). R: A language and environment for statistical computing (R Foundation for Statistical Computing).

Reid, J.E., and Wernisch, L. (2016). Pseudotime estimation: deconfounding single cell time series. Bioinformatics *32*, 2973–2980.

Sánchez-Danés, A., Richaud-Patin, Y., Carballo-Carbajal, I., Jiménez-Delgado, S., Caig, C., Mora, S., Di Guglielmo, C., Ezquerra, M., Patel, B., Giralt, A., et al. (2012). Disease-specific phenotypes in dopamine neurons from human iPS-based models of genetic and sporadic Parkinson's disease. EMBO Mol. Med. *4*, 380–395.

Sando, R., 3rd, Gounko, N., Pieraut, S., Liao, L., Yates, J., 3rd, and Maximov, A. (2012). HDAC4 governs a transcriptional program essential for synaptic plasticity and memory. Cell *151*, 821–834.

Sandor, C., Robertson, P., Lang, C., Heger, A., Booth, H., Vowles, J., Witty, L., Bowden, R., Hu, M., Cowley, S.A., et al. (2017). Transcriptomic profiling of purified patient-derived dopamine neurons identifies convergent perturbations and therapeutics for Parkinson's disease. Hum. Mol. Genet. *26*, 552–566.

Schöndorf, D.C., Aureli, M., McAllister, F.E., Hindley, C.J., Mayer, F., Schmid, B., Sardi, S.P., Valsecchi, M., Hoffmann, S., Schwarz, L.K., et al. (2014). iPSC-derived neurons from GBA1-associated Parkinson's disease patients show autophagic defects and impaired calcium homeostasis. Nat. Commun. *5*, 4028.

Soneson, C., Love, M.I., and Robinson, M.D. (2015). Differential analyses for RNA-seq: transcript-level estimates improve gene-level inferences. F1000Res. *4*, 1521.

Sternberg, C., Armstrong, A., Pili, R., Ng, S., Huddart, R., Agarwal, N., Khvorostenko, D., Lyulko, O., Brize, A., Vogelzang, N., et al. (2016). Randomized, double-blind, placebo-controlled phase III study of tasquinimod in men with metastatic castration-resistant prostate cancer. J. Clin. Oncol. *34*, 2636–2643.

Szewczyk-Krolikowski, K., Tomlinson, P., Nithi, K., Wade-Martins, R., Talbot, K., Ben-Shlomo, Y., and Hu, M.T. (2014). The influence of age and gender on motor and non-motor features of early Parkinson's disease: initial findings from the Oxford Parkinson Disease Center (OPDC) discovery cohort. Parkinsonism Relat. Disord. *20*, 99–105.

Takahashi, K., Tanabe, K., Ohnuki, M., Narita, M., Ichisaka, T., Tomoda, K., and Yamanaka, S. (2007). Induction of pluripotent stem cells from adult human fibroblasts by defined factors. Cell *131*, 861–872.

Tayebi, N., Walker, J., Stubblefield, B., Orvisky, E., LaMarca, M.E., Wong, K., Rosenbaum, H., Schiffmann, R., Bembi, B., and Sidransky, E. (2003). Gaucher disease with parkinsonian manifestations: does glucocerebrosidase deficiency contribute to a vulnerability to parkinsonism? Mol. Genet. Metab. *79*, 104–109.

Tolosa, E., Botta-Orfila, T., Morató, X., Calatayud, C., Ferrer-Lorente, R., Martí, M.J., Fernández, M., Gaig, C., Raya, Á., Consiglio, A., et al. (2018). MicroRNA alterations in iPSC-derived dopaminergic neurons from Parkinson disease patients. Neurobiol. Aging *69*, 283–291.

Van Wilgenburg, B., Browne, C., Vowles, J., and Cowley, S.A. (2013). Efficient, long term production of monocyte-derived macrophages from human pluripotent stem cells under partly-defined and fully-defined conditions. PLoS One *12*, e71098.

Young, M.D., Wakefield, M.J., Smyth, G.K., and Oshlack, A. (2010). Gene ontology analysis for RNA-seq: accounting for selection bias. Genome Biol. *11*, R14.

STAR★METHODS

KEY RESOURCES TABLE

REAGENT or RESOURCE	SOURCE	IDENTIFIER
Antibodies		
Tyrosine hydroxylase	Millipore	RRID: AB_90755
Beta-III tubulin (TUJ1)	Covance	RRID: AB_2313773
HDAC4	Abcam	RRID: AB_298903
β-actin	Abcam	RRID: AB_2305186
PDI	Cell signaling	RRID: AB_2156433
FKBP9	Abcam	RRID: AB_10562617
Ero1-Lα	Cell signaling	RRID: AB_823683
TSPAN7	Novus biologicals	RRID: AB_11035060
Na^{+}/K+-ATPaseα3 (ATP1A3)	Santa cruz	RRID: AB_10848453
Rtn1/2	Santa cruz	RRID: AB_2183564
PRKCB	ProSci	Cat#43-319
LAMP1	Santa cruz	RRID: AB_626853
LC3B	Sigma	RRID: AB_796155
TRA-1-60	Biolegend	RRID: AB_1186144
Nanog	Cell signaling	RRID: AB_10694485
Biological Samples		
OX1-19/SFC841-03-1/2	EBiSC	UOXFi004-B/ STBCi044-B
JR053-1/6	EBiSC	UOXFi005-A/ UOXFi005-B
AH016-3/6	EBiSC	University of Oxford
SFC156-03-01	EBiSC	STBCi101-A
SFC840-03-06	EBiSC	STBCi026-D
SFC067-03-01	EBiSC	STBCi105-A
MK088-1	EBiSC	UOXFi003-A
RH058-03	EBiSC	STBCi025-A/B/C
MK082-26	EBiSC	UOXFi002-A
MK071-3	EBiSC	UOXFi001-B
SFC077-03-04	EBiSC	STBCi268-A
SFC844-03-12	EBiSC	STBCi294-A
SFC120-03-04	EBiSC	STBCi043-B
SFC865-03-07	EBiSC	STBCi298-A
Chemicals, Peptides, and Recombinant Proteins		
ROCK inhibitor (Y27632 dihydrochloride)	Bio-Techne	Cat#1254
Tasquinimod	Tocris	Cat#S7617
Okadaic acid	Abcam	Cat#O7885
LB100	Tocris	Cat#S7537
Cantharidin	Tocris	Cat#1548
LDN-193189	Sigma	Cat#SML0559
SB-431542	Bio-Techne	Cat#1614
SHH C24II	Bio-Techne	Cat#1845-SH-500
Purmorphamine	Bio-Techne	Cat#4551/10
FGF8a	Stratech	Cat#16124-HNAE-SIB
CHIR-99021	Bio-Techne	Cat#4423
BDNF	Peprotech	Cat#450-02
GDNF	Peprotech	Cat#450-10

(*Continued on next page*)

Continued

REAGENT or RESOURCE	SOURCE	IDENTIFIER
TGFb3	Peprotech	Cat#100-36E
DAPT	Abcam	Cat#ab120633
Ascorbic acid	Sigma	Cat#A4544
(db)-cAMP	Sigma	Cat#D0627
hESC-qualified Matrigel	Corning	Cat#354277
DQ BSA Red	Thermo Fisher Scientific	Cat#D12051
NucBlue Live ReadyProbes	Thermo Fisher Scientific	Cat#R37605
Critical Commercial Assays		
Cytotune v1 Sendai Reprogramming kit	Thermo Fisher Scientific	A13780-01
Cytotune v2 Sendai Reprogramming kit	Thermo Fisher Scientific	A16517
β-Actin qPCR Control Kit	Eurogentec	SR-CL004-005
Human-HT-12-v4 expression BeadChip Kit	BD	BD-103-0204
All-Prep DNA/RNA Mini kit	QIAGEN	80204
RNeasy FFPE kit	QIAGEN	Cat#73504
RNA 6000 pico kit	Agilent	Cat#5067-1513
Quant-iT RiboGreen RNA kit	Thermo Fisher Scientific	Cat#R11490
Nextera XT DNA Library Prep Kit	Illumina	Cat#FC-131-1096
RNeasy Micro kit	QIAGEN	Cat#74004
Superscript III reverse transcriptase kit	Thermo Fisher Scientific	Cat#18080093
Fast SYBR green master mix	Thermo Fisher Scientific	Cat#4385612
αSyn extracellular release MSD kit	Meso Scale Discovery	Cat#K151TGD-2
Deposited Data		
Raw RNA-seq data	ArrayExpress	ArrayExpress: E-MTAB-7303
Software and Algorithms		
Harmony	Perkin Elmer	N/A
GenomeStudio	Illumina	N/A
Karyostudio	Illumina	N/A
TrimGalore v0.4.1	(Krueger, 2015)	https://www.bioinformatics.babraham.ac.uk/projects/trim_galore/
Kallisto v0.42.5	(Bray et al., 2016)	https://pachterlab.github.io/kallisto/
Picard 2.0.1	(Picard Toolkit, 2018)	https://broadinstitute.github.io/picard/
HISAT2	(Kim et al., 2015)	https://ccb.jhu.edu/software/hisat2/index.shtml
Tximport 1.4.0	(Soneson et al., 2015).	https://bioconductor.org/packages/release/bioc/html/tximport.html
Scater 1.8.0	(McCarthy et al., 2017)	https://bioconductor.org/packages/release/bioc/html/scater.html
Cellity 1.8.0	(Ilicic et al., 2016)	https://bioconductor.org/packages/release/bioc/html/cellity.html
DESeq2	(Love et al., 2014),	https://bioconductor.org/packages/release/bioc/html/DESeq2.html
goseq	(Young et al., 2010)	https://bioconductor.org/packages/release/bioc/html/goseq.html
The R project for statistical computing	R Development Core Team, 2008	https://www.r-project.org/
switchde 1.6.0	(Campbell and Yau, 2017)	https://bioconductor.org/packages/release/bioc/html/switchde.html
Ouija 0.99.0	(Campbell and Yau, 2017)	https://github.com/kieranrcampbell/ouija/
scran 1.8.2	(Lun et al., 2016)	https://bioconductor.org/packages/release/bioc/html/scran.html
Phenotypic Linkage Network	(Honti et al., 2014).	https://github.com/csandorfr/AP-PLN

CONTACT FOR REAGENT AND RESOURCE SHARING

Further information and requests for resources and reagents should be directed to and will be fulfilled by the Lead Contacts, Richard Wade-Martins (richard.wade-martins@dpag.ox.c.uk).

EXPERIMENTAL MODEL AND SUBJECT DETAILS

iPSC lines and participation recruitment

Participants were recruited to the Discovery clinical cohort through the Oxford Parkinson's Disease Centre and gave signed informed consent to mutation screening and derivation of iPSC lines from skin biopsies (Ethics committee: National Health Service, Health Research Authority, NRES Committee South Central, Berkshire, UK, REC 10/H0505/71). All the patients included in our study-fulfilled UK Brain Bank diagnostic criteria for clinically probable PD at presentation (Hughes et al., 1992). *GBA-N370S* PD patients 1, 2 and 4 presented with akinetic-rigid parkinsonism, and maintained a good levodopa-response for their first 5 years of treatment without significant falls or dementia. *GBA-N370S* patient 3 presented with akinetic-rigid parkinsonism, failed to respond to good doses of oral dopaminergic medication (600 mg levodopa, 150 mg benserazide daily), subsequently rapidly progressed more quickly with early dementia and frequent falls two years later. The patient has a revised diagnosis of Progressive Supranuclear Palsy (PSP). Patients with idiopathic Parkinson's who met the UK Parkinson's Disease Society Brain Bank (UKPDBB) criteria for the diagnosis of probable idiopathic PD (Hughes et al., 1992) on examination by a neurologist were recruited from ongoing cohort studies at the University of Oxford (UK) and the University of Lubeck (Germany) (Kasten et al., 2013). Patients with secondary parkinsonism due to head trauma or medication use, or features of atypical parkinsonism syndromes, were excluded (Szewczyk-Krolikowski et al., 2014).

Subject details

Donor ID	iPSC clone	Study ID	Genotype	Age & gende	Characterization
AH016	03/06	Control 1	wt/wt	80 M	Sandor et al., 2017
JR053	06/01	Control 2	wt/wt	68 M	This study
OX1 SFC841-03	19 01/02	Control 3	wt/wt	36 M	Van Wilgenburg et al., 2013
SFC156-03	01	Control 4	wt/wt	75 M	This study
SFC840-03	06	Control 5	wt/wt	67 F	Haenseler et al., 2017
SFC067-03	01	Control 6	wt/wt	72 M	This study
MK088	01	GBA 1	N370S/wt	46 M	Fernandes et al., 2016
MK071	03	GBA 2	N370S/wt	81 F	Fernandes et al., 2016
SFC834-03	03	GBA 3	N370S/wt	72 M	Fernandes et al., 2016
MK082	26	GBA 4	N370S/wt	51 M	This study
SFC077-03	04	Idiopathic PD 1	N/A	65 M	This study
SFC844-03	12	Idiopathic PD 2	N/A	72 M	This study
SFC120-03	04	Idiopathic PD 3	N/A	72 M	This study
SFC865-03	07	Idiopathic PD 4	N/A	69 M	This study

Culture, reprogramming and characterization of primary fibroblasts

Low passage fibroblast cultures were established from participant skin punch biopsies, and these were reprogrammed either by retroviral delivery or CytoTune-iPS Sendai Reprogramming kit (Thermo Fisher Scientific, version 1 or 2) as previously described (Fernandes et al., 2016). Clones were transitioned to feeder-free culture in mTeSR medium (StemCell Technologies), on hESC-qualified Matrigel-coated plates (BD), and passaged as cell clusters using 0.5 mM EDTA in PBS. Large batches were tested for mycoplasma (Mycoalert, Lonza), QCed (see below) and frozen at p15-25. When thawing for experiments, 10 μM ROCK inhibitor (Y27632, Bio-Techne) was added to promote initial survival and iPSC were passaged 1:2-3 using TryplE (Life Tech) with Y27632 during replating, culturing for maximum two weeks' post-thaw prior to differentiation to ensure consistency.

The following iPSC lines used in this study have been previously described: OX1-19 (van Wilgenburg et al., 2013), AH016-3/6 (Sandor et al., 2017), SFC840-03, MK088-1, MK071-3, SFC834-03 (Fernandes et al., 2016) and SFC840-03-06 (Haenseler et al., 2017). iPSC PD GBA lines, iPS MK082-26 and JR053-6, and idiopathic PD lines SFC077-03-04, SFC120-03-04, SFC844-03-12 and SFC865-03-07 are characterized here (Figure S1). Control lines SFC067-03-01 and SFC156-03-01 are registered in hPSCreg, with accompanying QC reports. Briefly, fluorescence activated cell sorting (FACS) for pluripotency markers TRA-1-60 (Biolegend) and Nanog (Cell Signaling) was performed on a FACSCalibur (BD Biosciences).

Silencing of retroviral delivered reprogramming genes was assessed by quantitative RT-PCR using the following primers: pMXsAS3200v2 TTA TCG TCG ACC ACT GTG CTG GCG mNanog forward primer GCT CCA TAA CTT CGG GGA GG. The β-Actin qPCR Control Kit (Eurogentec) was used as control normalization. Clearance of Cytotune Sendai vectors was performed by RT-PCR according to the manufacturer's instructions. Analysis of pluripotency gene expression profile was performed using the Human-HT-12-v4 expression BeadChip Kit (Illumina). Genome integrity was assessed applying the Illumina Human CytoSNP-12v2.1 beadchip array or Illumina human OmniExpress24 on genomic DNA generated using the All-Prep kit (QIAGEN) and analyzed using GenomeStudio and Karyostudio software (Illumina).

Generation and characterization of iPSC derived dopamine neurons

Six control (OX1-19/SFC841-03-01/02, JR053-6/1, AH016-3/6, SFC156-03-01, SFC840-03-06 and SFC067-03-01), four *GBA-N370S* (MK088-1, MK071-3, SFC834-03-03 and MK082-26) patient lines and four idiopathic (SFC077-03-04, SFC844-03-12, SFC120-03-04 and SFC865-03-07) patient lines were differentiated, as described previously (Kriks et al., 2011), with slight modifications (Beevers et al., 2017). Cells underwent 21 days of patterning and differentiation, were replated and matured for a further 5 weeks (60 DIV) when collected for flow cytometry. Control and PD *GBA-N370S* patient lines were successfully differentiated into dopaminergic neurons, expressing beta-tubulin III (*TUJ1*) a neuronal marker and Tyrosine Hydroxylase (*TH*) a specific dopaminergic neuronal marker by immunofluorescence (Figure S2A). Treatment of iPSC-derived dopamine neurons with the HDAC4 modifying compounds occurred at DIV 45 for 72 hours at the following concentrations: Tasquinimod (15uM), Okadaic acid (10nM), LB100 (1.25uM) and Cantharidin (250nM).

METHOD DETAILS

Purification of iPSC dopaminergic neurons by flow cytometry

Purification of iPSC derived dopaminergic neurons was carried out as previously described (Sandor et al., 2017). At sorting each sample was first sorted into each row of a 96 well plate, into 2 ul of smart-seq 2 lysis buffer (0.2% triton x-100 and 2 U/ul RNase inhibitor), so that all samples were on each 96 well plate for single cell RNA-sequencing. After 96 well plate sorting, the rest of the sample was bulk collected for RNA extraction in preparation for bulk RNA-sequencing.

RNA preparation of bulk RNA-seq samples

RNA from bulk collected FACS sorted dopamine neurons was extracted using an FFPE RNA extraction kit (QIAGEN) as per manufacturer's instructions, with minor modifications. RNA integrity analysis was analyzed using a 2100 bioanalyzer system and a RNA 6000 pico kit (Agilent). Concentration was obtained and confirmed utilizing two methods; the 2100 bioanalyzer system and a Quant-iT RiboGreen RNA kit (Invitrogen), as per manufacturer's instructions.

Smart-seq2, RNA library construction and sequencing

Single cells and RNA extracted from bulks were processed using the Smart-seq2 protocol (Picelli et al., 2013). cDNA samples were prepared for sequencing using the Nextera XT DNA Library Prep Kit (Illumina) with our own in-house indexing primers (Lamble et al., 2013). Libraries were pooled and sequenced using Illumina HiSeq4000 75bp paired-end sequencing.

RNA-seq read alignment and expression quantification

Single-cell and bulk RNA-seq data was processed identically. FASTQ files were trimmed using TrimGalore v0.4.1 on default settings. Transcript expression levels were quantified using Kallisto v0.42.5 (Bray et al., 2016) against GRCh38 reference human transcriptome. Additional quality control (QC) metrics were compiled by Picard 2.0.1 (https://broadinstitute.github.io/picard/) on BAM files aligned to the human genome (GRCh38) using Hisat2 (Kim et al., 2015). Transcript level abundances were then summarized to gene level estimates using tximport 1.4.0 (https://bioconductor.org/packages/release/bioc/html/tximport.html) (Soneson et al., 2015).

Quality control of single-cell RNA-seq

Quality-control, visualization, and handling of single-cell data was performed using Scater (McCarthy et al., 2017). Cells belonging to plates 3-6 were removed due to distinct clustering on reduced-dimensionality representations and low expression of otherwise constitutively expressed genes. Further outliers were removed using Cellity (Ilicic et al., 2016) and subsequently any cell expression *GAPDH* at a level below the maximum *GAPDH* expression in blank wells was further removed, leaving a total of 146 cells for analysis.

Differential expression analysis

Differential expression analysis on bulk RNA-seq was performed using DESeq2 (Love et al., 2014), including a covariate to account for technical replicates. GO enrichment was performed using goseq (Young et al., 2010) and over-represented p values were multiple test corrected using the Benjamini-Hochberg procedure.

Single-cell pseudotime analysis

A PCA representation of the cells was computed using the prcomp function in the stats package in R using the 500 most variable genes (in log expression space), the default in Scater. Single-cell differential expression analysis along PC2 was performed using the R package switchde (Campbell and Yau, 2017). A further refined trajectory using the combined gene list along was computed using Ouija.

Identification of pathway activation in GBA 3

Over-dispersion analysis was performed in the method identical to Brennecke et al. (2013) using the R package scran (Lun et al., 2016) using ERCC spike-ins. A gene was designated as over-dispersed if the reported q-value < 0.05. GO analysis was performed using the R package GOSeq (Young et al., 2010). Genes from the *SRP-dependent co-translational protein targeting to membrane* pathway were selected for validation by performing a Wilcoxon rank-sum test for log2(TPM+1) expression in GBA3 cells compared to controls and prioritized based on p value.

Phenotypic linkage network construction

To assess functional similarity and convergence of the core gene set we constructed a phenotypic linkage network (Honti et al., 2014). We wished to assess the functional similarity of genes within the core set compared to a randomly sampled background distribution. The genes selected for the background distribution should match the overall expression pattern of the core set in these iPSCs in order to account for the increased likelihood of functional similarities between genes randomly selected from the same cell type. We first noted that the core set of genes exhibited high mean expression than average among the whole transcriptome.

We then fitted a gamma distribution to the mean log2(TPM+1) values of both the core gene set and all other genes in the transcriptome (the core distribution and background distribution). Then for each gene not in the core gene set we calculated the probability of observing the mean expression level under both models, and formed an unnormalized inclusion probability of the ratio of the density of the observed expression given the core gene set distribution to the density of the observed expression given the background distribution. This can loosely be thought of as "how many times more likely is it that a gene fits the core set distribution compared to the background distribution." To choose the background set of genes we then sampled 1000 genes from the transcriptome excluding the core gene set, where the probability of a gene being selected was proportional to the un-normalized inclusion probability. The resulting empirical distribution fitted well with the fitted core gene set distribution (Figure S4D). We subsequently constructed a phenotypic linkage network as per Honti et al. (2014) using 1) the core set of genes, 2) the 1000 sampled background genes, and 3) the genes *SNCA, PARK2, PARK7, LRRK2, UCHL1, GBA, PINK1, ATP13A2, HTRA2, PLA2G6, VPS35*, and *EIF4G1*. Links were compared between different classes of genes using a one-sided Wilcoxon rank-sum test.

qRT-PCR, immunocytochemistry and western blot

For qRT-PCR experiments to validate RNA-Seq findings RNA was extracted from 12 well plates using Trizol (life technologies) and purified using the RNeasy Micro kit (QIAGEN) as per manufacturer's instructions. Quality and concentration were quantified using a Nanodrop 1000 (Thermo Scientific). cDNA was synthesized using a superscript III reverse transcriptase kit (Life technologies) as per manufacturer's instructions. qRT-PCR was carried out using fast SYBR green mastermix and a StepOnePlus thermal cycler (Life technologies). Primers used in this study can be found in Table S2.

For immunocytochemistry cells were fixed in 4% paraformaldehyde in 96 well plates (microClear 96 well plates, Greiner). They were then blocked with 10% donkey serum (PBS/0.5% triton x) for 1 hour, incubated with the following antibodies; Tyrosine hydroxylase (1:500 Millipore AB1542), Beta-III tubulin (1:500 Covance MMS 435P), HDAC4 (1:500 Abcam ab12171) and DAPI in 1% donkey serum (PBS/0.5% triton x). Secondary antibodies were added in 1% donkey serum (PBS) for 1 hour. Cells were washed and kept in 1x PBS for imaging on the Opera Phenix (Perkin Elmer).

For western blotting cells were extracted from 12 well plates in RIPA buffer (Tris [50 mM, pH 8], sodium chloride [150 mM], sodium dodecyl sulfate [SDS; 0.1% w/v], sodium deoxycholate [0.5% w/v] and nonidet-P40 [1% w/v]). Samples were denatured for 5 minutes at 100°C. Protein separation was achieved using SDS polyacrylamide gel electrophoresis and transferred onto PVDF membrane. Antibodies used as follows: Tyrosine hydroxylase (1:500 Millipore AB1542), β-actin (1:10,000 Abcam ab8227), PDI (1:500 Cell signaling 3501), FKBP9 (1:500 Abcam ab91219), Ero1-Lα (1:500 Cell signaling 9956), TSPAN7 (1:250 Novus Biologicals NBP1-90310), Na^{+}/K^{+}-ATPase α3 (1:500 Santa cruz sc-365744), Rtn1/2 (1:500 Santa cruz sc-71981), PRKCB (1:250 ProSci 43-319), LAMP1 (1:500 Santa cruz sc-20011), LC3B (1:500 Sigma L7543), HDAC4 (1:500 Abcam ab12171).

DQ-BSA

DQ-BSA Red reagent was prepared according to manufacturer's instructions. While remaining under treatment of the selected compounds, iPSC-derived dopaminergic neurons were incubated with 30mg/mL DQ BSA Red and NucBlue Live ReadyProbes reagents for 4 hours at 37°C. Cells were washed with DPBS, replaced into fresh media and imaged on the Opera Phenix High Content Screening System (Perkin Elmer).

α-synuclein release

Extracellular α-synuclein release was quantified as previously described (Fernandes et al., 2016). Briefly, conditioned media from treated iPSC-derived neuronal cultures (100 μl) was collected at D45 and stored at −80°C prior to analysis. α-synuclein release

was quantified relative to a standard curve using an electrochemiluminescent assay (Meso Scale Discovery, MD, USA, Cat# K151TGD-2) and a MESO QuickPlex SQ 120 instrument (Meso Scale Discovery). Extracellular α-synuclein release was normalized relative to total protein content of cells, using a BCA assay.

QUANTIFICATION AND STATISTICAL ANALYSIS

For differences between more than two groups a two way-ANOVA analysis was used to test for the significance. Mean values ± SEM are shown unless otherwise stated. P value for comparisons were adjusted for multiple comparisons using a Bonferroni correction. Data was presented of 3 independent controls and patients, over three differentiations unless otherwise stated.

Cell Stem Cell, Volume *24*

Supplemental Information

Single-Cell Sequencing of iPSC-Dopamine Neurons Reconstructs Disease Progression and Identifies HDAC4 as a Regulator of Parkinson Cell Phenotypes

Charmaine Lang, Kieran R. Campbell, Brent J. Ryan, Phillippa Carling, Moustafa Attar, Jane Vowles, Olga V. Perestenko, Rory Bowden, Fahd Baig, Meike Kasten, Michele T. Hu, Sally A. Cowley, Caleb Webber, and Richard Wade-Martins

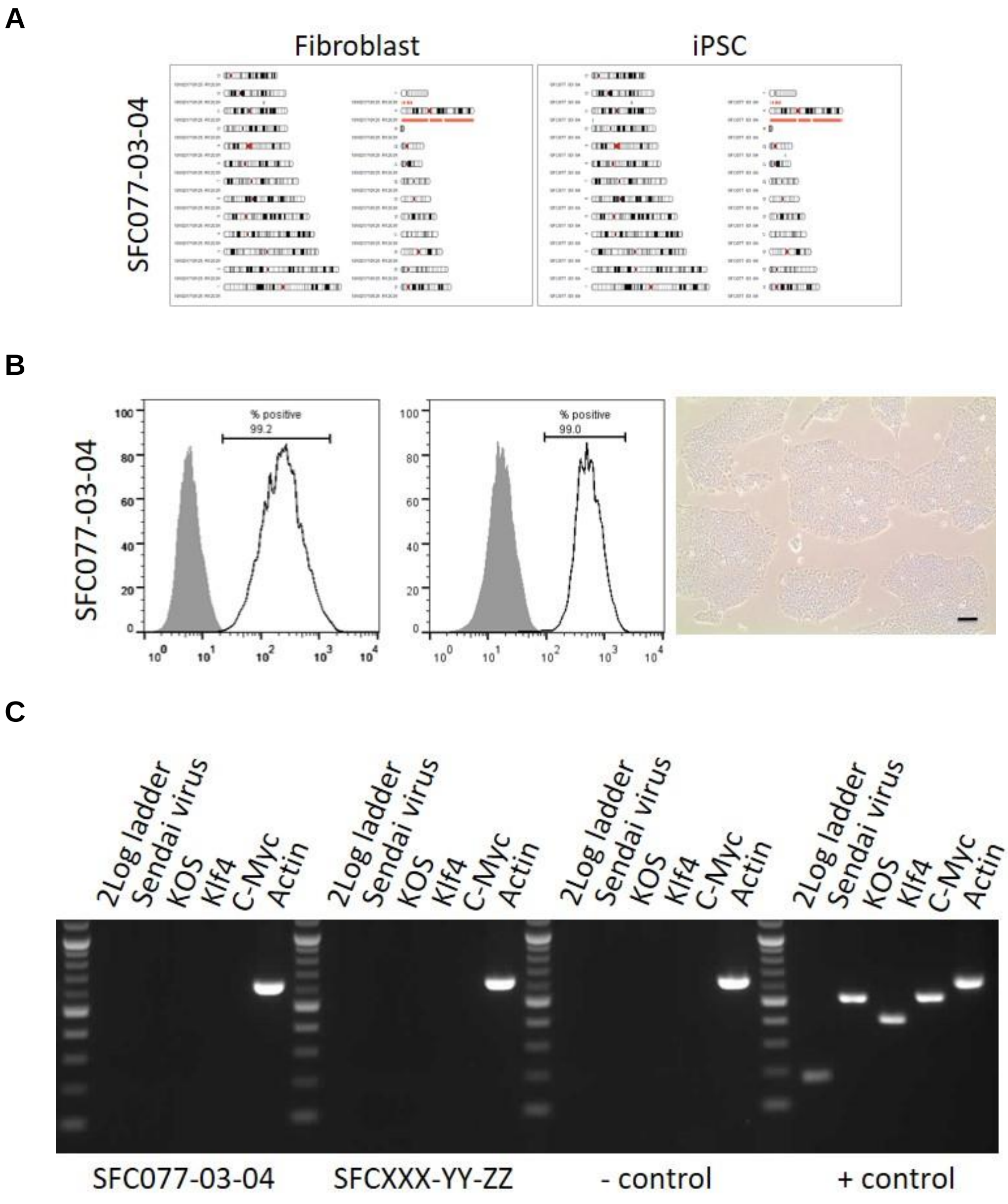

Supplementary Figure 1. Quality control (QC) of iPSC lines used in this study (related to STAR methods section Culture, reprogramming and characterisation of primary fibroblasts).
Representative iPSC QC of line SFC077-03-04 **(A)** Genome integrity was assessed by Illumina Human CytoSNP-12v2.1 or OmniExpress24SNP array and karyograms produced using KaryoStudio software (Illumina). Amplifications (green)/deletions (orange)/LOH regions (grey) are shown alongside the relevant chromosome (NB single-copy sex chromosomes are annotated orange). **(B)** FACS analysis confirmed expression of pluripotency markers Tra-1-60 and Nanog in iPSCs; open black plot represents antibody, filled grey plot represents isotype control; Right-hand panel shows expected iPSC colony morphology by phase-contrast microscopy. Scale bar = 100 µm. **(C)** Clearance of Cytotune Sendai vectors from iPSC lines: top panel Cytotune 1 (Log2 ladder; Sendai backbone 181 bp; Sox2 451 bp; Klf4 410 bp; c-myc 532 bp; Oct-4 483 bp; β-actin control 92 bp; + control, fibroblasts infected with Cytotune 5 days previously); lower panels Cytotune 2 (as per Cytotune 1 PCR products, but with KOS 528bp, β-actin control 623 bp).
All previously unpublished lines used in this paper have been deposited in the European Bank for induced pluripotent Stem Cells, EBiSC, https://cells.ebisc.org/ and are listed in the Human pluripotent stem cell registry, hPSCreg, https://hpscreg.eu/ along with their QC data.

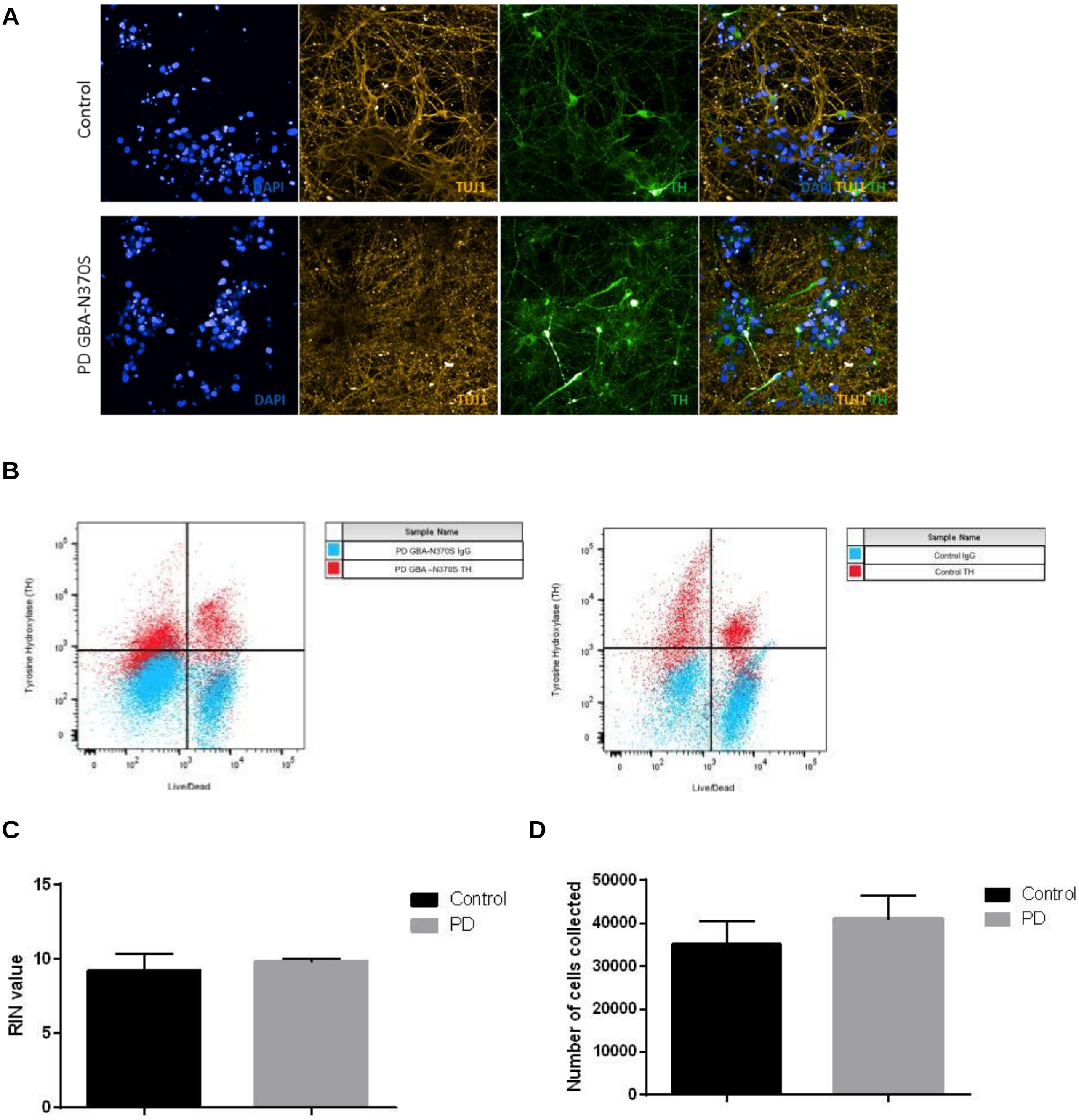

Supplementary Figure 2. Immunofluorescence, fluorescence activated cell sorting (FACS) and RNA integrity (RIN) values of Control and PD *GBA-N370S* samples (related to STAR methods section Generation and characterisation of iPSC derived dopamine neurons).
(A) Representative images of the successful differentiation of control and PD *GBA-N370S* dopaminergic neurons, displaying the nuclear marker (DAPI), neuronal marker (TUJ1) and the dopaminergic marker (TH). **(B)** Representative FACS plots of sorted TH positive neurons from Control and PD *GBA-N370S* samples. IgG2a was used as a FACS antibody control. **(C)** Number of TH+ cells collected from 3 control and 3 PD *GBA-N370S* samples. **(D)** RIN values of bulk sorted RNA from 3 control and 3 PD *GBA-N370S* samples.

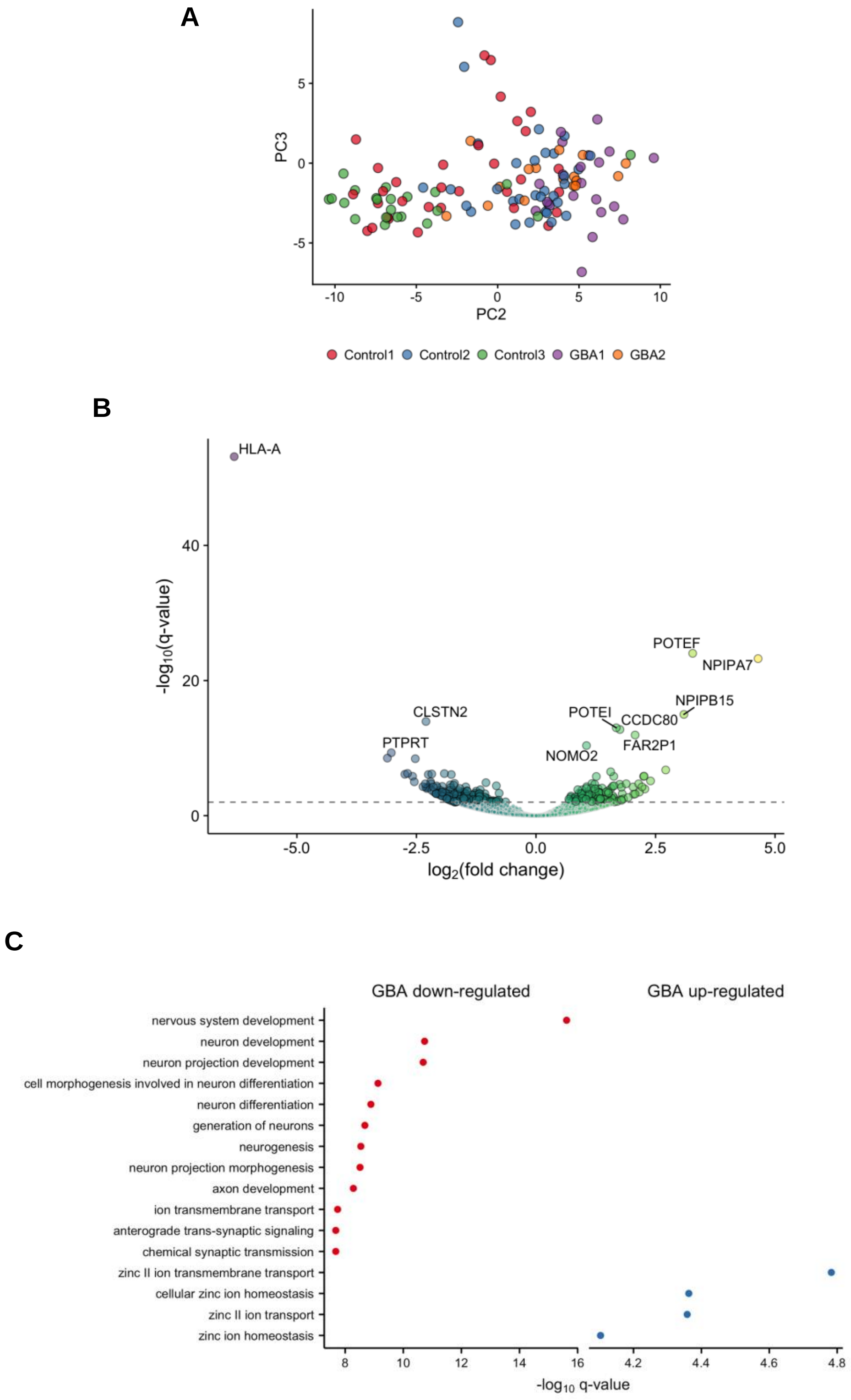

Supplementary Figure 3. Bulk and single DE genes analysis with the removal of GBA3 (related to Figure 2).

(A) Transcriptome PCA analysis after the removal of GBA3, resolving the remaining two PD *GBA-N370S* PD patients and three controls. **(B)** Volcano plot of the differential expression analysis using DESeq2 identified 310 genes (FDR 1%) differentially expressed between case-control with the removal of GBA3 **(C)** A GO enrichment analysis of the up and down-regulated genes in PD *GBA-N370S* patients, with the removal of GBA3, highlights important pathways involved in zinc homeostasis and neuronal function respectively.

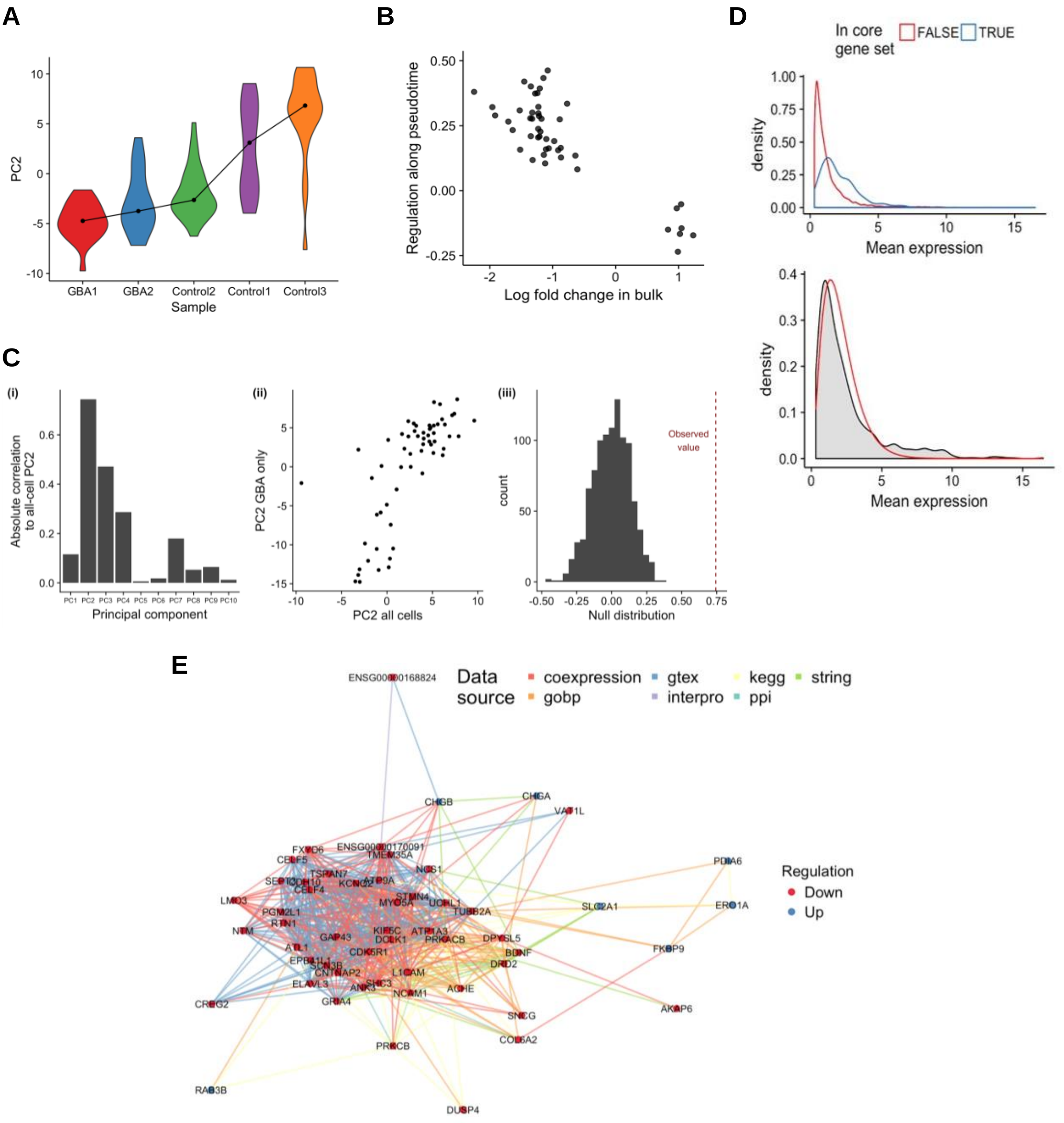

Supplementary Figure 4. Single-cell trajectory analysis recapitulates a case-control ordering of cells and Bulk and single cell RNA-seq combined gene set analysis (related to Figure 3).
(A) Violin plot showing principal component 2 (PC2) scores for each sample after the removal of GBA3. PC2 roughly segregates cells into case/control status and thus may be seen to represent the continuous transcriptional signature of cells from control to GBA status. **(B)** The regulation along pseudotime (PC2) as provided by *switchde* show consistent regulation with bulk DE analysis for the core gene set identified in both single and bulk DE analysis along with SC3 clustering. **(C-i)** PC2 of the data is equivalent in GBA samples alone, demonstrating the control to disease transition heterogeneity exists within the disease samples. **(C-ii)** The correlation of PC2 using GBA cells only compared to the top 10 PC’s using all cells reveals high correlation with PC2 (rho = 0.74), exemplified in a scatter plot. **(C-iii)** By randomly permuting cells, performing PCA and comparing correlations to PC2, a null distribution was created, the observed correlation $p < 0.001$. **(D)** Mean expression of genes in the core gene set compared to whole transcriptome and compared to Gamma distribution fit (red). **(E)** A phenotypic linkage network (PLN) representing functional similarity between the core set of genes identified in both bulk and single-cell RNA-seq.

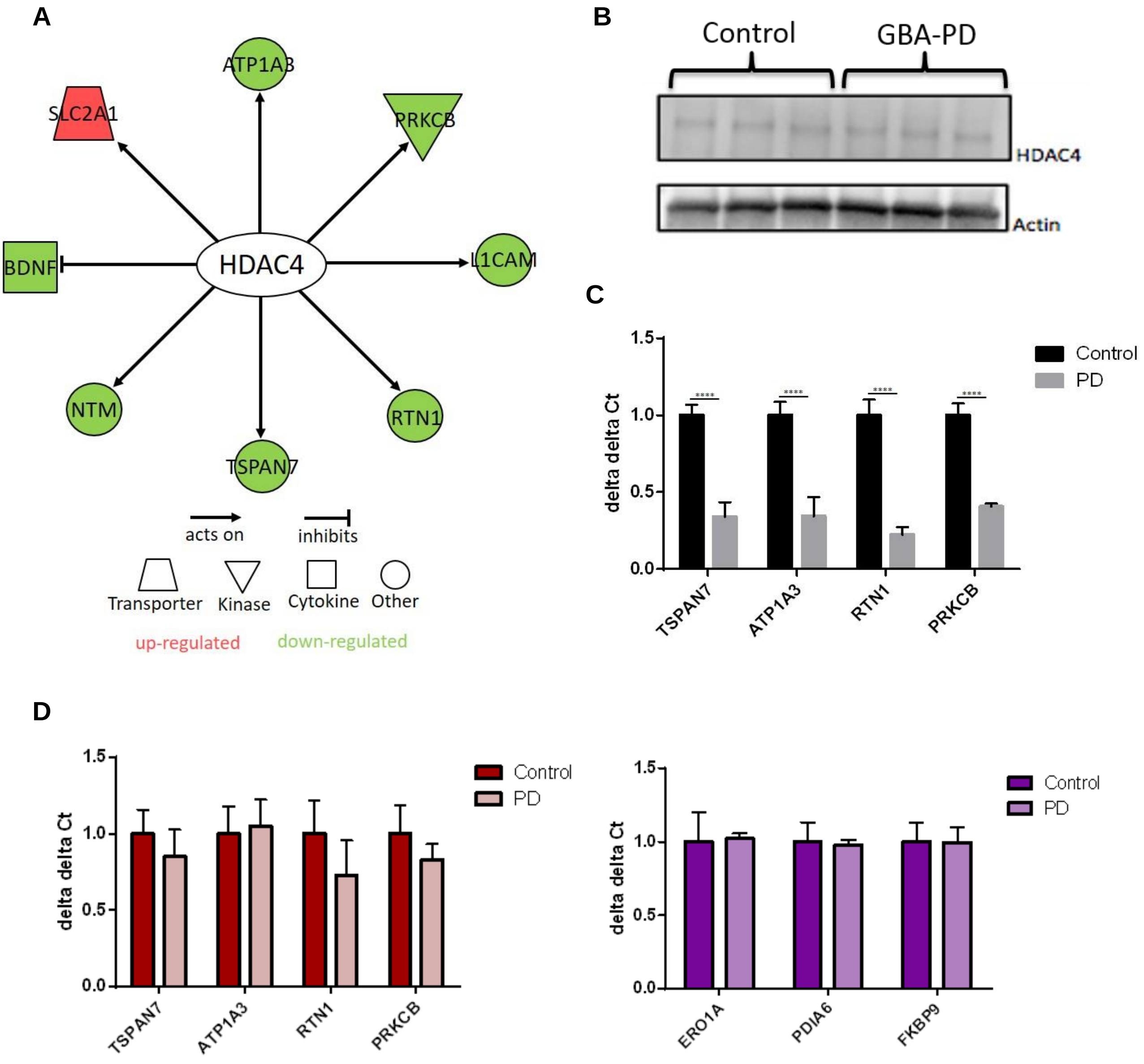

Supplementary Figure 5. HDAC4 as a master regulator of a set of down-regulated genes in PD *GBA-N370S* patients (related to Figure 3).

(A) IPA (Qiagen) identified HDAC4 as an upstream regulator of a number of genes identified as down-regulate and one up-regulated gene, in the core set of 60 DE genes (modified from IPA Qiagen). **(B)** HDAC4 protein levels are unchanged in control and PD *GBA-N370S* patients. **(C)** The four HDAC4 controlled genes in the core set of genes identified in both bulk and single cell RNA-seq were confirmed as down-regulated in PD *GBA-N370S* patients compared to controls by qRT-CPR (****p<0.0001). **(D)** No difference in the expression of HDAC4 controlled genes (*TSPAN7*, *ATP1A3*, *RTN1* and *PRKCB*) and ER stress genes (*ERO1A*, *PDIA6* and *FKBP9*) identified in the case-control axis, in induced pluripotent stem cells pre-differentiation.

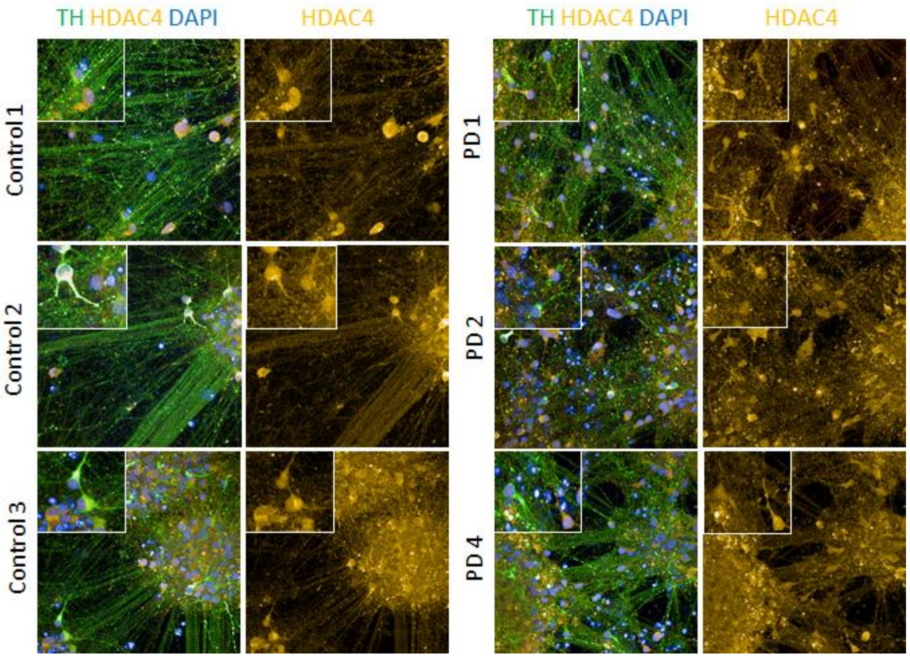

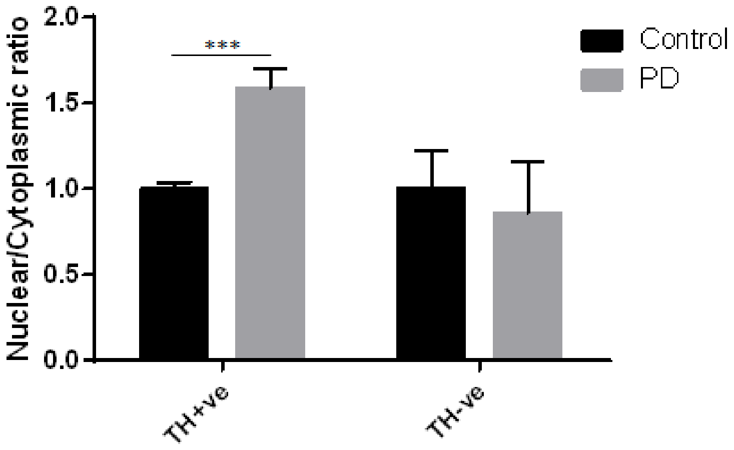

Supplementary Figure 6. HDAC4 nuclear vs cytoplasmic ratio in non-dopaminergic neurons in PD *GBA-N370S* patients and controls (related to Figure 4).

No difference in HDAC4 nuclear/cytoplasmic ratio intensity in non-TH positive neurons in three control and three PD *GBA-N370S* patient lines. Data represented as mean ± SEM ***p<0.001 of three control and three PD *GBA-N370S* patients.

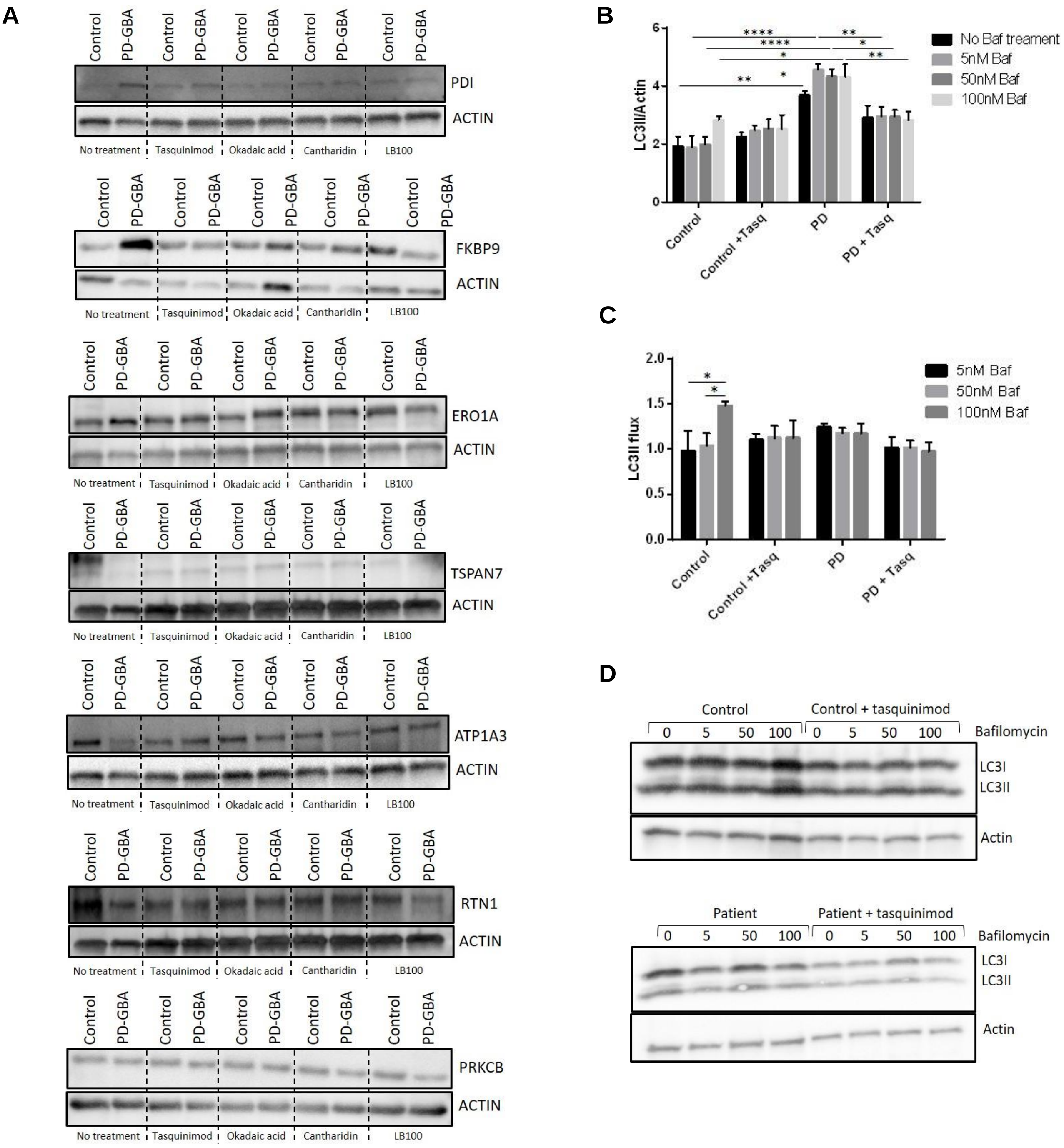

Supplementary Figure 7. Representative western blot images of the protein graphs in Figure 5 and autophagic flux in control and PD *GBA-N370S* patient iPSC-derived dopamine neurons with and without tasquinimod treatment (related to Figure's 5 and 6).

(A) Western blots display an increase in ER stress proteins (*PDI*, *FKBP9* and *ERO1A*) and a decrease in HDAC4 related proteins (*TSPAN7*, *ATP1A3*, *RTN1* and *PRKCB*) in PD *GBA-N370S* patients compared to controls. The addition of the HDAC4 modulating drugs (Tasquinimod, Okadaic acid, Cantharidin and LB100) mostly revert these phenotypes. **(B)** LC3II/Actin levels in control and PD *GBA* neurons +/- tasquinimod and 0, 5, 50 and 100 nM bafilomycin. Black bars (no bafilomycin treatment) and grey bars (with bafilomycin treatment). **(C)** Data in Figure 1A represented as autophagic flux, quantified by dividing levels of LC3-II with bafilomycin treatment by the level of LC3-II without treatment. **(D)** Representative western blots for LC3-II and actin in control and PD *GBA* neurons, compared to a calibration sample. Data represented as mean ± SEM (*p<0.05, **p<0.01 and ****p<0.0001) of three control and three PD *GBA-N370S* patients.

Supplementary Table 1. Control, PD *GBA-N370S* and Idiopathic PD lines used in this study (related to STAR methods section iPSC lines and participation recruitment and subject details table).

Donor ID	iPSC clone	Study ID	Genotype	Age	Characterisation
AH016	03/06	Control 1	wt/wt	80 M	Sandor et al 2017
JR053	06/01	Control 2	wt/wt	68 M	This study
OX1<br>SFC841-03	18/19<br>01/02	Control 3	wt/wt	36 M	Van Wilgenburg et al 2013
SFC156-03	01	Control 4	wt/wt	75 M	This study
SFC840-03	06	Control 5	wt/wt	67 F	Fernandes et al 2016
SFC067-03	01	Control 6	wt/wt	72 M	This study
MK088	01	GBA 1	N370S/wt	46 M	Fernandes et al 2016
MK071	03	GBA 2	N370S/wt	81 F	Fernandes et al 2016
SFC834-03	03	GBA 3	N370S/wt	72 M	Fernandes et al 2016
MK082	26	GBA 4	N370S/wt	51 M	This study
SFC077-03	04	Idiopathic PD 1	N/A	65 M	This study
SFC844-03	12	Idiopathic PD 2	N/A	72 M	This study
SFC120-03	04	Idiopathic PD 3	N/A	72 M	This study
SFC865-03	07	Idiopathic PD 4	N/A	69 M	This study

Supplementary Table 2. QRT-PCR primers used in this study (related to STAR methods section qRT-PCR, immunocytochemistry and western blot).

Primer name	Primer sequence	Source
B2M F	TTCTGGCCTGGAGGCTATC	This paper
B2M R	TCAGGCAATTTGACTTTCCATTC	This paper
FKBP9 F	AGCTTGCCTACGGAAATGAA	This paper
FKBP9 R	GGGGCTTGAAATAGGTGTGA	This paper
ERO1A F	AATGTCGTCTGTGGGGAAAG	This paper
ERO1A R	AGGTCCACTTTCTGGCATATTT	This paper
PDIA6 F	CAGAATGGAAGAAAGCAGCA	This paper
PDIA6 R	TCCCTGAACACCATACTGACC	This paper
TSPAN7 F	TGTTGTCTTTGGCCTGTTTG	This paper
TSPAN7 R	TGACGAAACACAAACCCTGA	This paper
ATP1A3 F	CCTTGGAGACTCGGAACATC	This paper
ATP1A3 R	GATACGGCCCATGACAGTG	This paper
RTN1 F	AGTGCAGAAAACCGACGAAG	This paper
RTN1 R	GGAAGAGCCTCCTCAGTTCC	This paper
PRKCB F	TACTCCAGCCCCACGTTTTG	This paper
PRKCB R	TCACTTCCTTCTGGTGGCAC	This paper
RPS12 F	TGCGTTCAAGATTCAACTTCAC	This paper
RPS12 R	TGAGGGCAGTCTTCAGAACC	This paper
RPS17 F	CATTATCCCCAGCAAAAAGC	This paper
RPS17 R	CCTCTTACTGGGCCTCTCTG	This paper
RPS6 F	CGATGAACGCAAACTTCGTA	This paper
RPS6 R	ACCACTGATTCGGACCACAT	This paper
pMXsAS3200v2	TTATCGTCGACCACTGTGCTGGCG	(Takahashi et al., 2007)
mNanog	GCTCCATAACTTCGGGGAGG	(Takahashi et al., 2007)
